# Novel Models of Image Permutation and Diffusion Based on Perturbed Digital Chaos

**DOI:** 10.3390/e22050548

**Published:** 2020-05-13

**Authors:** Thang Manh Hoang, Safwan El Assad

**Affiliations:** 1 School of Electronics and Telecommunications, Hanoi University of Science and Technology, 1 Dai Co Viet, Hai Ba Trung, Hanoi 100000, Vietnam; 2 IETR (Institut d’Electronique et des Télécommunications de Rennes), Université de Nantes, CNRS, UMR 6164, Polytech Nantes, Rue Christian Pauc CS 50609, CEDEX 3, 44306 Nantes, France; safwan.elassad@univ-nantes.fr

**Keywords:** chaos-based image encryption, chaotic cryptography, dynamics perturbation, chaotic permutation, chaotic diffusion

## Abstract

Most of chaos-based cryptosystems utilize stationary dynamics of chaos for the permutation and diffusion, and many of those are successfully attacked. In this paper, novel models of the image permutation and diffusion are proposed, in which chaotic map is perturbed at bit level on state variables, on control parameters or on both. Amounts of perturbation are initially the coordinate of pixels in the permutation, the value of ciphered word in the diffusion, and then a value extracted from state variables in every iteration. Under the persistent perturbation, dynamics of chaotic map is nonstationary and dependent on the image content. The simulation results and analyses demonstrate the effectiveness of the proposed models by means of the good statistical properties of transformed image obtained after just only a single round.

## 1. Introduction

For recent decades, chaos has been discovered in natural, human, and engineering models [1]. It has been also generated by human for pragmatic applications. Two of prominent applications are chaotic communications [2] and chaos-based cryptography [3]. Recently, chaos-based image encryption has attracted increasing interest [4,5,6,7]. That is due to the good cryptographic properties of chaotic sequences [8,9,10,11] and a chaotic system can be implemented on digital hardware [12,13,14,15]. In digital hardware, dynamics of any chaotic system is degraded to periodic orbits due to the round-off errors by the limited number of bits represented for values of state variables and control parameters [16,17,18]. The larger the number of bits representing for chaotic state variable and control parameters is, the longer the length of period is obtained. Beside that, the period of orbits produced by a chaotic map can be lengthened by several methods as suggested in Reference [19]. Two of such methods are perturbation on chaotic states by another chaotic map [20,21] and by using linear feedback shift register (LFSR) [22].

For chaos-based cryptography, at least one of encryption processes is involved by chaos. Along with the Feistel structure, the substitution-permutation network (SPN) structure attains the properties of confusion and diffusion [23], which are widely employed in both conventional block ciphers [24,25] and chaotic ones [26,27,28,29]. Typically, the SPN structure can be realized in chaotic ciphers by means of the combination of permutation and diffusion processes, for example, References [30,31]. The advantage of the SPN structure is that the cryptographic statistics can be increased by means of increasing the number of rounds in each of permutation and diffusion processes and/or in a whole.

For most of chaos-based image cryptosystems, a chaotic system is used for generating chaotic sequences for the permutation and diffusion processes. Firstly, the chaotic permutation is implemented with the involvement of at least one chaotic system to shuffle pixels or bits of pixels within the image. The permutation rule can be static in the form of table or dynamic by inducing from chaotic values. Secondly, the chaotic diffusion is usually realized by a mixture between chaotic values and values of plain pixels. In literature, most of successful attacks on chaotic ciphers are based on weaknesses in algorithms of permutation and diffusion processes, for example, References [32,33,34,35,36,37]. Besides, the works [38,39] points out the criteria and assessment to a chaotic cryptosystem.

Under a cryptographic point of view, it is obviously that the more complicated dynamics of chaos allows the stronger chaos-based cryptosystem. Recently, many chaos-based cryptosystems were proposed with the use of more complicated chaos. Along with the use of hyperchaotic, time-delay, fractional order, and spatiotemporal chaotic systems, complicated dynamics can be obtained by mixed of various chaotic systems such as References [40,41,42,43,44,45]. In such the chaos-based cryptosystems, chaotic systems work with fixed values of control parameters and with non-disturbed chaotic orbits. In other words, dynamics of chaotic maps is stationary in generating encryption keys for the permutation and diffusion.

It is also well-known that analysis of chaotic dynamics can be performed by the observation and measurement of dynamics like trace formulas [46,47,48,49] or inference of control parameters [50,51,52], and so forth. Many analysis methods success with additive perturbation [46,47,48]. With the development of analysis methods, analysis of chaotic dynamics can used as a powerful tool to attack chaos-based cryptosystems [53]. However, to date, there has not been any report about a successful attack to a chaotic block cipher by means of analysis of chaotic dynamics. By applying analysis of chaotic dynamics, chaotic cryptosystems based on stationary dynamics will become possibly insecure in the future. Therefore, one of potential approaches of chaotic image encryption is based on perturbed chaos.

Definitely, a chaos-based cryptosystem becomes much stronger if its encryption keys are dependent on the image content. The involvement of image content in chaotic dynamics is created by an external perturbation. In fact, there are two approaches to create the connection between the image content and encryption keys, dependent on whether the image content involves in chaotic dynamics or not. Firstly, the connection between the image content and encryption keys is established by means of state perturbation, for example, References [9,42,54,55,56,57,58]. References [9,54] present the a selection mechanism in which the image content is used for selecting one of chaotic sequences to generate keystreams. The initial values of chaotic system are fixed, and neither state variables nor control parameters of chaotic system is disturbed during generation of chaotic sequences. As presented in Reference [42], the initial value and the value of parameter of chaotic system are generated with the use of image content for whole encryption, but the parameter of chaotic map deciding the manner of the permutation and diffusion is dependent on the image content of blocks. The advantage is that the value of parameter of chaotic system is updated after every block of image. As presented in Reference [55], the initial value of chaotic map in the diffusion process is calculated by the value of pixels, and the output of chaotic map is used to compute the ciphertext. The important point is that the image content involves in the diffusion by means of its initial value of chaotic map. Whereas, the value of control parameters is kept constant. The same approach as given in References [55,59] is used in the work by G. Ye et al. [56], in which the initial value of chaotic map is computed by information entropy of plain image. In Reference [59], the diffusion process utilizes one of state variables of hyper-chaotic Chen’s system, in which only initial value of chaotic map is being updated after every pixel. In the work of H. Li et al. [58], the orbit of two-dimensional logistic-adjusted-sine map (2D-LASM) is disturbed by the coordinate and the value of pixels during the generation of the keystreams for the permutation and diffusion, while the value of control parameters of (2D-LASM) is kept constant. In another way, the initial value of chaotic map is the output of authentication by SHA-256 as in Reference [57], or the value of control parameter of chaotic map is calculated by the image content as in Reference [60] for generation of finite state machines for the diffusion. As reported in Reference [61], the value of control parameter of Logistic map is calculated by the image content, and it is kept constant in the encryption process. The common point in those works is that control parameters of chaotic maps are unperturbed, so dynamics of chaotic maps is stationary.

Secondly, the dependence of encryption keys on the image content can be created by perturbing on control parameters of chaotic systems. To the best of our knowledge, there are a limited number of published works in this way as in References [62,63]. Specifically, in the work proposed by J. Chen et al. [62], the control parameter of Logistic map is perturbed in the pixel swapping confusion and diffusion processes. In the work by T. Song [63], the control parameter of Logistic map is computed by using the value of pixels, and updated in the diffusion process. In fact, the disadvantage is that the value range of control parameter must be always monitored and adjusted under a condition. Moreover, a number of additional arithmetic operations along with those of chaotic map requires higher computational complexity and resource.

In addition to two main approaches as above described, some other works presents the utilization of perturbed chaos for cryptosystems, for example, References [21,62,64,65]. In References [21,64,65], the chaotic maps are perturbed by additional transformations of state variables or by some conditions, rather than by information of pixels. In fact, additional equations and conditions make chaotic maps more mathematically complicated, but not really perturbed by any external force. Thus, dynamics of chaotic maps is stationary, and the vulnerability still exists [66].

Overall, perturbed dynamics of chaos with the dependence on the image content offers the cryptographic properties better than those with stationary dynamics in terms of statistics and it can resist from the type of chosen plaintext attack. However, reported image cryptosystems based on perturbed chaos have the proprietary structures with the use of specific chaotic systems, and perturbation is realized in arithmetic operations. Under the viewpoint of hardware, more arithmetic operations will require more resource and may reduce the speed of the encryption. In other words, there is a lack of models with a simpler perturbation than those in previous works, utilization of various chaotic maps, and suitability for hardware implementation.

In this paper, novel models of perturbed digital chaos are proposed for the image permutation and diffusion. Perturbation on chaotic dynamics is carried out at bit level by three schemes, that is, perturbation on state variables, on control parameters and on both state variables and control parameters (“on both” for short). Amounts of perturbation can be either the coordinate of pixels in the permutation and the value of pixels in the diffusion or the value extracted from state variables. Chaotic dynamics becomes nonstationary and it provides cryptographic advantages for the image permutation and diffusion. The example and simulation results demonstrate the effectiveness of the proposed models with the use of Logistic map. It is noted that this work will not go into analysis of dynamic properties of chaotic systems under perturbation, and ones can find that in other works, for example, References [19,46,67,68], and so forth.

The main contributions of the work are as follows: The structures of chaotic perturbation with an external force are generalized, in which three schemes of perturbation are clearly expressed. The models of the permutation and diffusion for the chaotic image encryption are proposed by means of utilizing the corresponding schemes of perturbation. The perturbation is the coordinate of pixels in the permutation and the value of pixels in the diffusion. The statistical and security analyses are carried out for the example using the generic Logistic map as a proof of effectiveness of the proposed models.

The rest of the paper is organized as follows—Section 2 presents some basic preliminaries. The general structures of perturbed chaotic map are given in Section 3. Next, the proposed models of permutation and diffusion for chaotic image encryption are detailed in Section 4. Section 5 shows the example and the simulation results for the permutation and diffusion with various schemes of perturbation using the Logistic map. Finally, Section 6 gives some concluding remarks of the work.

## 2. Some Basic Preliminaries

### 2.1. Representation of Images

Let us consider the raster format of a grayscale image is represented as a 2-dimensional matrix *I* with the size M×N. The element of *I* at the location (x,y) is called a pixel P(x,y) in binary of *k* bits as $P_{XY}=b_{k-1}b_{k-2}\ldots b_{1}b_{0}$. The image can be considered as a collection of pixels represented by

(1) $$\begin{array}{c} I=\overset{M-1}{\underset{x=0}{⋃}}\overset{N-1}{\underset{y=0}{⋃}}P\left(x,y\right), \end{array}$$

where *M* and *N* are the number of rows and columns of pixels, respectively. In the following text, an entity formed by a collection of elements is denoted by ⋃ with an associated index. In the case of RGB image, each of three color layers can be considered as a grayscale image.

### 2.2. Bit Representation for Real Numbers

**Figure 1:**
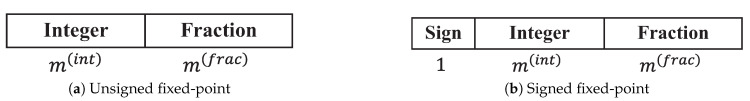
Representation of fixed-point number.

Let us consider that a chaotic map in Equation (6) is implemented in a digital platform. So, the value of state variables and that of control parameters are represented in one of two formats, that is, fixed-point or floating-point number. Fixed-point representation is suitable for most chaotic maps because the value of state variables and of control parameters are in the narrow ranges. Signed and unsigned fixed-point numbers are illustrated in Figure 1.

For example, the Logistic map has the range of (0,1) for chaotic state variable, and that of [3.57,4.0] for the control parameter, thus, the format of unsigned fixed-point is suitable. The number of bits required for the integer part in the value of chaotic state and of control parameter are 1 and 3, respectively.

For a signed fixed-point representation, a real number is represented one bit for the sign *S*, $m^{\left(int\right)}$ bits for the integer part and $m^{\left(frac\right)}$ bits for the fractional part; or $m=1+m^{\left(int\right)}+m^{\left(frac\right)}$. The fixed-point number can be written in sequence of bits as $\left(S\right)b_{m^{\left(int\right)}-1}\ldots b_{0}\left(.\right)b_{-1}\ldots b_{-m^{\left(frac\right)}}$. Note that the binary point is in the parentheses, ‘(’ and ‘)’. The value is $V={\left(-1\right)}^{S}\sum_{i=-m^{\left(frac\right)}}^{m^{\left(int\right)}-1}b_{i}\times 2^{i}$.

For a unsigned fixed-point representation, there is no sign bit. Thus, the number of bits is $m=m^{\left(int\right)}+m^{\left(frac\right)}$; representation in bit sequence is $b_{m^{\left(int\right)}-1}\ldots b_{0}\left(.\right)b_{-1}\ldots b_{-m^{\left(frac\right)}}$; and the value is $V=\sum_{i=-m^{\left(frac\right)}}^{m^{\left(int\right)}-1}b_{i}\times 2^{i}$.

As a real number is represented as a bit sequence, bitwise operations can be applied to change the state of bits.

### 2.3. Representation of Bit Sequence and Bit Arrangement

Here, the bit arrangement is to permute bits, but the term “bit arrangement” is used to avoid confusing the “permutation” of pixels in the later part of the paper. Let us consider two arrays of bit sequences $A={\left(A_{i}\right)}_{1\leq i\leq I_{A}}$ and $B={\left(B_{i}\right)}_{1\leq i\leq I_{B}}$. Bit sequences of *A* and *B* are $A_{i}={⋃}_{j=1}^{J_{A}}a_{i,j}$ and $B_{i}={⋃}_{j=1}^{J_{B}}b_{i,j}$, respectively. There, $a_{i,j}$ and $b_{i,j}$ are $j^{th}$ bits of $i^{th}$ sequences, and $J_{A}$ and $J_{B}$ are the lengths of bit sequences $A_{i}$ and $B_{i}$, respectively. In order to simplify for the representation, the size of *A* and *B* is denoted by $I_{A}\times J_{A}$ and $I_{B}\times J_{B}$, respectively.

Let us define a bit arrangement for a general case of $I_{A}\neq I_{B}$ and $J_{A}\neq J_{B}$. Bit sequences of *A* are constructed by bits from sequences of *B*. The rule of bit arrangement is encoded by a matrix *Y*, and the arrangement operator is denoted by ∘, such that A=Y∘B. For the matrix $Y={\left[y_{i,j}\right]}_{1\leq i\leq I_{A},\;1\leq j\leq J_{A}}$, $y_{i,j}$ is the combination of indexes indicating a bit of *B*. Each row of *Y*, $Y_{i}={\left[y_{i,j}\right]}_{\;1\leq j\leq J_{A}}$, is used for constructing a bit sequence $A_{i}$, in other words, bit sequences of *A* are $A_{i}=Y_{i}\circ B$. It is noted that a bit $b_{i,j}$ of *B* can be used multiple times in *A*.

For example, the array of bit sequences *B* has the size of $\left(I_{B},J_{B}\right)=\left(5,4\right)$ as

(2) $$B=\left(\begin{array}{c} b_{1,1}b_{1,2}b_{1,3}b_{1,4}\\ b_{2,1}b_{2,2}b_{2,3}b_{2,4}\\ b_{3,1}b_{3,2}b_{3,3}b_{3,4}\\ b_{4,1}b_{4,2}b_{4,3}b_{4,4}\\ b_{5,1}b_{5,2}b_{5,3}b_{5,4} \end{array}\right).$$

The array *A* is constructed from bits of *B*. *A* is with three bit sequences, and each sequence has six bits; or the size of *A* is $\left(I_{A},J_{A}\right)=\left(3,6\right)$. The matrix *Y* is

(3) $$Y=\left[\begin{array}{cccccc} \left(4,3\right) & \left(1,4\right) & \left(2,3\right) & \left(4,1\right) & \left(2,2\right) & \left(3,1\right)\\ \left(1,3\right) & \left(3,4\right) & \left(3,2\right) & \left(2,1\right) & \left(5,4\right) & \left(3,2\right)\\ \left(4,3\right) & \left(3,2\right) & \left(1,4\right) & \left(3,1\right) & \left(5,3\right) & \left(2,2\right) \end{array}\right].$$

So, the array *A* is as

(4) $$A=Y\circ B=\left(\begin{array}{c} b_{4,3}b_{1,4}b_{2,3}b_{4,1}b_{2,2}b_{3,1}\\ b_{1,3}b_{3,4}b_{3,2}b_{2,1}b_{5,4}b_{3,2}\\ b_{4,3}b_{3,2}b_{1,4}b_{3,1}b_{5,3}b_{2,2} \end{array}\right),$$

where, three bit sequences are $A_{1}=b_{4,3}b_{1,4}b_{2,3}b_{4,1}b_{2,2}b_{3,1}$, $A_{2}=b_{1,3}b_{3,4}b_{3,2}b_{2,1}b_{5,4}b_{3,2}$ and $A_{3}=b_{4,3}b_{3,2}b_{1,4}b_{3,1}b_{5,3}b_{2,2}$.

If a certain bit of $A_{i}$ is fixed with a predefined state ’0’ or ’1’, the terms $BIT_{0}$ and $BIT_{1}$ are used to indicate the states ‘0’ and ‘1’ in $Y_{i}$, respectively. For instance, the value of bits in *A* is fixed such as $A_{1}=\left(1\right)b_{1,4}b_{2,3}b_{4,1}b_{2,2}\left(0\right)$, so $Y_{1}$ must be as $Y_{1}=\left[BIT_{1},\left(1,4\right),\left(2,3\right),\left(4,1\right),\left(2,2\right),BIT_{0}\right]$.

Moreover, except for a number of bits with fixed values, let us define $H_{Y}$ to be the number of bits in *A* taken from *B* as

(5) $$H_{Y}=\sum_{i=1}^{I_{A}}\sum_{j=1}^{J_{A}}y_{i,j}\;\;\;\;\;\;\;\forall y_{i,j}\notin \left(BIT_{0},BIT_{1}\right).$$

These will be used in the proposed models of permutation and diffusion in the later part of the paper.

## 3. Perturbed Digital Chaotic Map

It is definitely that dynamics of a chaotic system becomes nonstationary if the chaotic system is perturbed by an external force. In this section, a chaotic map with perturbation at bit level is described in two primitive schemes, that is, perturbation on state variables and on control parameters of chaotic map. The third scheme is the combination of two mentioned ones, in which both state variables and control parameters of chaotic map are perturbed. In any scheme, dynamics of chaotic system becomes complicated and that brings advantages in terms of cryptographic properties.

Let us consider a chaotic map defined by

(6) $$\left\{\begin{array}{c} X_{n+1}=F\left(X_{n},\Gamma \right),\\ X_{n}=\left[x_{n}^{\left(D\right)}\;\;x_{n}^{\left(D-1\right)}\ldots \;x_{n}^{\left(2\right)}\;\;x_{n}^{\left(1\right)}\right],\\ \Gamma_{n}=\left[\gamma_{n}^{\left(G\right)}\;\;\gamma_{n}^{\left(G-1\right)}\ldots \;\gamma_{n}^{\left(2\right)}\;\gamma_{n}^{\left(1\right)}\right], \end{array}\right.$$

where $X_{n}$ and $\Gamma_{n}$ are vectors of chaotic state variables and of control parameters, respectively; *D* is the number of dimensions, and *G* is the number of control parameters; $D=\left|\right|X_{n}\left|\right|$ and $G=\left|\right|\Gamma_{n}\left|\right|$. The perturbation on state variables is

(7) $$X_{n+1}=F\left({\hat{X}}_{n},\Gamma_{0}\right),$$

on control parameters is

(8) $$X_{n+1}=F\left(X_{n},{\hat{\Gamma }}_{n}\right),$$

and on the both of state variables and control parameters is

(9) $$X_{n+1}=F\left({\hat{X}}_{n},{\hat{\Gamma }}_{n}\right).$$

There, ${\hat{X}}_{n}$ and ${\hat{\Gamma }}_{n}$ are the perturbed variables and control parameters, respectively described as

(10) $$\left\{\begin{array}{cc} {\hat{X}}_{n} & =\Psi_{X}\left(X_{n},\Delta_{X}\right),\\ \Delta_{X} & =\Omega_{X}\left(X_{n},E_{X}\right),\\ \Delta_{X} & ={\left[\delta_{X}^{\left(D\right)}\;\;\delta_{X}^{\left(D-1\right)}\ldots \;\;\delta_{X}^{\left(2\right)}\;\;\delta_{X}^{\left(1\right)}\right]}^{T}, \end{array}\right.$$

and

(11) $$\left\{\begin{array}{cc} {\hat{\Gamma }}_{n} & =\Psi_{\Gamma }\left(\Gamma_{n},\Delta_{\Gamma }\right),\\ \Delta_{\Gamma } & =\Omega_{\Gamma }\left(X_{n},E_{\Gamma }\right),\\ \Delta_{\Gamma } & ={\left[\delta_{\Gamma }^{\left(G\right)}\;\;\delta_{\Gamma }^{\left(G-1\right)}\ldots \;\;\delta_{\Gamma }^{\left(2\right)}\;\;\delta_{\Gamma }^{\left(1\right)}\right]}^{T}. \end{array}\right.$$

There, ${\hat{X}}_{n}$ and ${\hat{\Gamma }}_{n}$ are ${\hat{X}}_{n}=\left[{\hat{x}}_{n}^{\left(D\right)}\;\;{\hat{x}}_{n}^{\left(D-1\right)}\ldots \;{\hat{x}}_{n}^{\left(2\right)}\;\;{\hat{x}}_{n}^{\left(1\right)}\right]$ and ${\hat{\Gamma }}_{n}=\left[{\hat{\gamma }}_{n}^{\left(G\right)}\;{\hat{\gamma }}_{n}^{\left(G-1\right)}\ldots \;{\hat{\gamma }}_{n}^{\left(2\right)}\;{\hat{\gamma }}_{n}^{\left(1\right)}\right]$, respectively; $\Delta_{X}$ and $\Delta_{\Gamma }$ are instant amounts of perturbation; $\Psi_{X}$ and $\Psi_{\Gamma }$ define the operations of perturbation; $\Omega_{X}=\left\{\omega_{X}^{\left(i\right)},i=\left\{1,\ldots ,D\right\}\right\}$ and $\Omega_{\Gamma }=\left\{\omega_{\Gamma }^{\left(i\right)},i=\left\{1,\ldots ,G\right\}\right\}$ are sets of functions to produce amounts of perturbation; $E_{X}$ and $E_{\Gamma }$ are vectors of external forces. Note that, the subscripts *X* and Γ denote for the notations belonging to state variables and control parameters, respectively.

In hardware perspective, values of state variables and control parameters are represented in a format of real number. Thus, all of functions, that is, $\Omega_{X}$, $\Omega_{\Gamma }$, $\Psi_{X}$, and $\Psi_{\Gamma }$, operate at bit level. Specifically, at bit level, each bit of operands in such the functions can be manipulated by basic logic gates AND, OR, NOR, NAND, NOT, XOR, XNOR or their combination.

**Figure 2:**
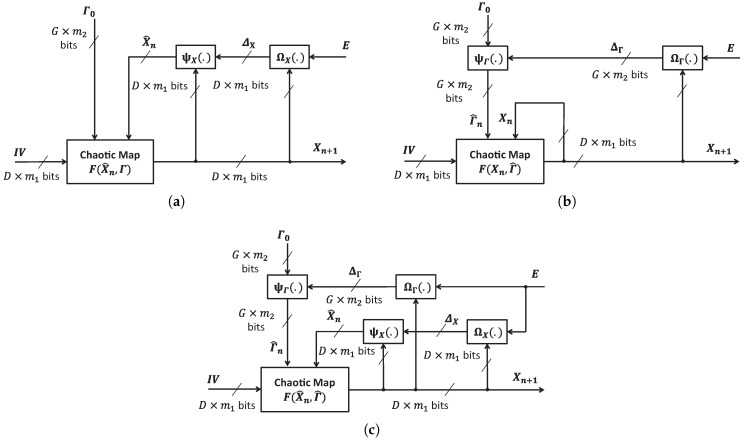
Chaotic map with perturbations (**a**) on state variables, (**b**) control parameters, and (**c**) both of state variables and control parameters.

Figure 2 illustrates the proposed schemes of perturbation. The perturbation can be on chaotic state variables, control parameters, or both. IV is a vector of initial condition.

In any scheme, perturbation must ensure that chaos exhibits and values of $X_{n}$ and $\Gamma_{n}$ must be within valid ranges. As given in Equations (10) and (11), the value ranges of $X_{n}$ and $\Gamma_{n}$ are dependent on both amounts and functions of perturbation. At bit level, values of $X_{n}$ and $\Gamma_{n}$ are represented in the format of fixed point as shown in Section 2.2. Therefore, $\Delta_{X}$ and $\Delta_{\Gamma }$ define the perturbation to $X_{n}$ and $\Gamma_{n}$, respectively. The disturbance level on a chaotic map is really dependent on the position of perturbed bits in the representation.

Also, values of ${\hat{X}}_{n}$ and ${\hat{\Gamma }}_{n}$ are represented by a number of bits, and specific position of perturbed bits is pointed out by perturbation functions $\Psi_{X}\left(.\right)$ and $\Psi_{\Gamma }\left(.\right)$. Let us $\Theta_{X}$ and $\Theta_{\Gamma }$ respectively be vectors of value tolerances of state variables and control parameters, $\Theta_{X}=\left|{\hat{X}}_{n}-X_{n}\right|$ and $\Theta_{\Gamma }=\left|{\hat{\Gamma }}_{n}-\Gamma_{n}\right|$. Equivalently, at each time of perturbation, ${\hat{X}}_{n}$ and ${\hat{\Gamma }}_{n}$ in Equations (7)–(9) are

(12) $${\hat{X}}_{n}=X_{n}\pm \Theta_{X},$$

and,

(13) $${\hat{\Gamma }}_{n}=\Gamma_{n}\pm \Theta_{\Gamma },$$

where, $\Theta_{X}=\left\{\theta_{X}^{\left(i\right)},i=\left\{1,\ldots ,D\right\}\right\}$ and $\Theta_{\Gamma }=\left\{\theta_{\Gamma }^{\left(i\right)},i=\left\{1,\ldots ,G\right\}\right\}$. As described above, values of $\theta_{X}^{\left(i\right)}$ and $\theta_{\Gamma }^{\left(i\right)}$ are dependent on the state of bits in $\delta_{X}^{\left(i\right)}$ and $\delta_{\Gamma }^{\left(i\right)}$ making bits in $x_{n}^{\left(i\right)}$ and $\gamma_{n}^{\left(i\right)}$ changed. The value ranges of $\theta_{X}^{\left(i\right)}$ and $\theta_{\Gamma }^{\left(i\right)}$ can be figured out when the positions of perturbed bits are known in a specific scheme of perturbation. In general, this can be defined by ones, and the larger amounts of perturbation will make the more complexity in chaotic dynamics. This suggests that the higher significant bits of $X_{n}$ and $\Gamma_{n}$ should be perturbed.

As shown in Equations (10) and (11), the amounts of perturbation, $\Delta_{X}$ and $\Delta_{\Gamma }$, are dependent on pairs of values ($X_{n}$,$E_{X}$) and ($X_{n}$,$E_{\Gamma }$), respectively. At bit level, all functions of $\Omega_{X}$ and $\Omega_{\Gamma }$ are bitwise operations, thus basic logic gates and their combination can be used for bit manipulation.

## 4. Proposed Models of Permutation and Diffusion

In this section, the models of permutation and diffusion are proposed those are based on the proposed schemes of perturbation as described in the previous Section. It is noted that the XOR operation is chosen as the function of perturbation. The superscripts (p) and (d) are associated on the notations to indicate the permutation and diffusion.

### 4.1. Proposed Chaotic Pixel Permutation (CPP) with Perturbation

**Figure 3:**
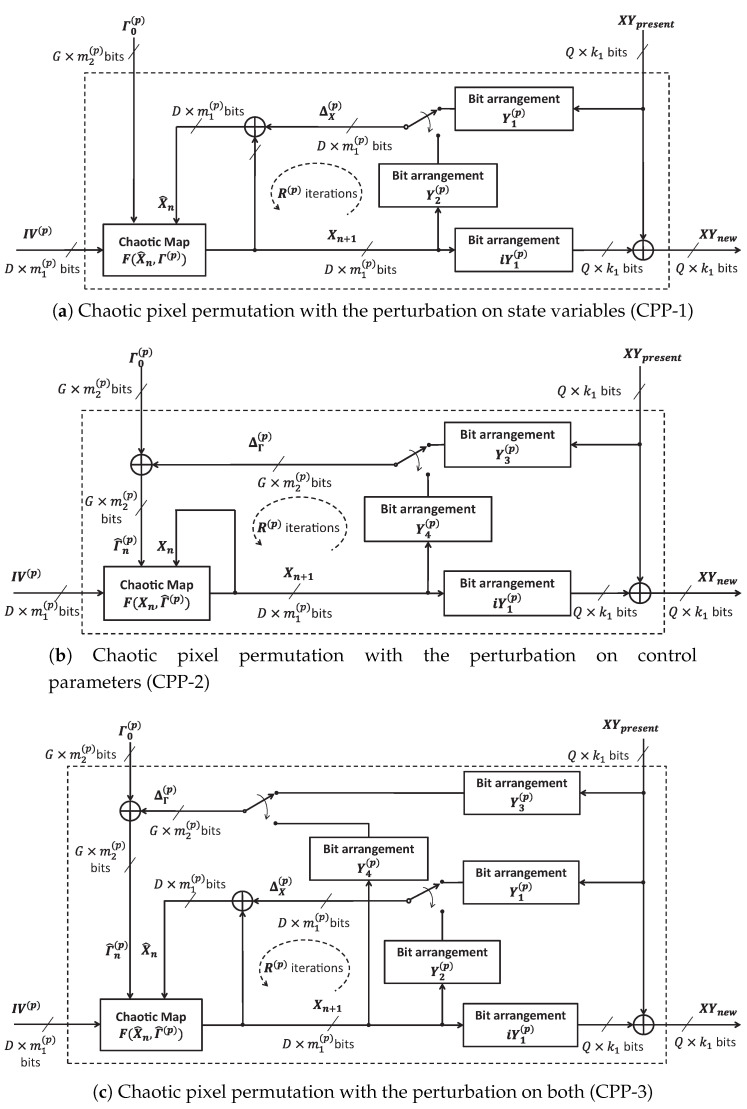
The structure of Chaotic Pixel Permutations (CPPs) with the perturbation.

Pixel permutation shuffles pixels within the space of image using chaos. The idea of bit-level perturbation to chaotic map as illustrated in Section 3 is employed to propose three configurations of CPP as illustrated in Figure 3. The perturbation to a chaotic system is carried out on state variables (CPP-1), on control parameters (CPP-2), and on both (CPP-3) as in Figure 3a, Figure 3b, and Figure 3c, respectively.

It is assumed that a *D*-dimensional chaotic map F(.) has *G* control parameters. Values of state variables and control parameter are represented in the fixed-point format by $m_{1}^{\left(p\right)}$ and $m_{2}^{\left(p\right)}$ bits, respectively. So, values of state variables $X_{n}$ and its perturbation $\Delta_{X}^{\left(p\right)}$ can be seen as arrays of bit sequences with the size of $D\times m_{1}^{\left(p\right)}$. Similarly, values of control parameters $\Gamma_{n}^{\left(p\right)}$ and its perturbations $\Delta_{\Gamma }^{\left(p\right)}$ are represented by arrays of bit sequences with the size of $G\times m_{2}^{\left(p\right)}$ bits. Bit arrangements $Y_{1}^{\left(p\right)}$, $iY_{1}^{\left(p\right)}$, $Y_{2}^{\left(p\right)}$, $Y_{3}^{\left(p\right)}$, and $Y_{4}^{\left(p\right)}$ are to arrange the size of arrays of bit sequences as described Section 2.3. The size of inputs and outputs is given in Table 1.

In chaotic behavior, there are constraints in the value ranges of chaotic state variables and control parameters. Specifically, the constraints are met by fixing a number of bits in chaotic state variables and in control parameters, while the rest number of bits can be changeable by the perturbation. So, the number of bits $Q\times m_{1}^{\left(p\right)}$ representing for the coordinate of pixels $XY_{present}$ and $XY_{new}$ must be less than the number of changeable bits in all the schemes of perturbation. For simplest case, the coordinate of pixels is encoded by a sequence of $k_{1}$ bits, in which row and column numbers of pixels are respectively represented by $k_{1}^{\left(x\right)}$ and $k_{1}^{\left(y\right)}$ bits; $k_{1}=k_{1}^{\left(x\right)}+k_{1}^{\left(y\right)}$.

The XOR operation is chosen as the perturbation functions $\Psi_{X}$ and $\Psi_{\Gamma }$ in Equations (10) and (11). In this paper, bit arrangements play a role of the sets of functions $\Omega_{X}\left(.\right)$ and $\Omega_{\Gamma }\left(.\right)$ generating amounts of perturbation $\Delta_{X}^{\left(p\right)}$ and $\Delta_{\Gamma }^{\left(p\right)}$, respectively. For the CPP-1, the chaotic map is perturbed by means of modification of bits in chaotic state variables $X_{n}$ with bits in an amount of perturbation $\Delta_{X}^{\left(p\right)}$ after every iteration *n* ($1\leq n\leq R^{\left(p\right)}$), while the value of control parameters $\Gamma^{\left(p\right)}$ is kept constant. Therefore, the deterministic orbit of chaotic map is destroyed by such the perturbation amount $\Delta_{X}^{\left(p\right)}$. Similarly, the value of control parameters of chaotic map $\Gamma^{\left(p\right)}$ are changed after every iteration in the CPP-2. Under the perturbation on control parameters, dynamics of chaotic map becomes nonstationary. The CPP-3 is the combination of the CPP-1 and CPP-2 that both state variables and control parameters are updated after every iteration.

Respectively, the state variables of chaotic map with the perturbation as given in Figure 3a–c are

(14) $$\begin{array}{c} \left\{\begin{array}{cc} {\hat{X}}_{0}=IV^{\left(p\right)}⊕\Delta_{X}^{\left(p\right)},\\ X_{n+1}=F\left({\hat{X}}_{n},\Gamma_{0}^{\left(p\right)}\right) & \;\mathrm{for}\;n=\{1\ldots R^{\left(p\right)}\}, \end{array}\right. \end{array}$$

(15) $$\begin{array}{c} \left\{\begin{array}{cc} X_{0}=IV^{\left(p\right)},\\ {\hat{\Gamma }}_{0}^{\left(p\right)}=\Gamma_{0}^{\left(p\right)}⊕\Delta_{\Gamma }^{\left(p\right)},\\ X_{n+1}=F\left(X_{n},{\hat{\Gamma }}_{n}^{\left(p\right)}\right) & \;\mathrm{for}\;n=\{1\ldots R^{\left(p\right)}\}, \end{array}\right. \end{array}$$

(16) $$\begin{array}{c} \left\{\begin{array}{cc} {\hat{X}}_{0}=IV^{\left(p\right)}⊕\Delta_{X}^{\left(p\right)},\\ {\hat{\Gamma }}_{0}^{\left(p\right)}=\Gamma_{0}^{\left(p\right)}⊕\Delta_{\Gamma }^{\left(p\right)},\\ X_{n+1}=F\left({\hat{X}}_{n},{\hat{\Gamma }}_{n}^{\left(p\right)}\right) & \;\mathrm{for}\;n=\{1\ldots R^{\left(p\right)}\}. \end{array}\right. \end{array}$$

The perturbed state variables and control parameters are as

(17) $$\begin{array}{c} {\hat{X}}_{n}=X_{n}⊕\Delta_{X}^{\left(p\right)},\\ {\hat{\Gamma }}_{n}^{\left(p\right)}=\Gamma_{n}^{\left(p\right)}⊕\Delta_{\Gamma }^{\left(p\right)}. \end{array}$$

Amounts of perturbation are represented in arrays of bit sequences $\Delta_{X}^{\left(p\right)}$ and $\Delta_{\Gamma }^{\left(p\right)}$ after bit arrangements as

(18) $$\begin{array}{c} \Delta_{X}^{\left(p\right)}=\left\{\begin{array}{cc} Y_{1}^{\left(p\right)}\circ XY_{present} & \mathrm{for}\;n=1;\\ Y_{2}^{\left(p\right)}\circ X_{n} & \mathrm{for}\;2\leq n\leq R^{\left(p\right)}, \end{array}\right. \end{array}$$

and

(19) $$\begin{array}{c} \Delta_{\Gamma }^{\left(p\right)}=\left\{\begin{array}{cc} Y_{3}^{\left(p\right)}\circ XY_{present} & \mathrm{for}\;n=1;\\ Y_{4}^{\left(p\right)}\circ X_{n} & \mathrm{for}\;2\leq n\leq R^{\left(p\right)}, \end{array}\right. \end{array}$$

After $R^{\left(p\right)}$ iterations, the value of $X_{R^{\left(p\right)}}$ is used to obtain the new coordinate of pixels as

(20) $$XY_{new}=XY_{present}⊕\left(iY_{1}^{\left(p\right)}\circ X_{R^{\left(p\right)}}\right).$$

It is noted that ∘ is the bit arrangement as given in Section 2.3.

### 4.2. Inverse Chaotic Pixel Permutation

Let us consider Inverse Chaotic Pixel Permutation (iCPP) as shown in Figure 3. The present coordinate of pixels is converted into bits sequence $XY_{present}$, and the XOR operation is used to produce new position, $XY_{new}$, at the last iteration. Therefore, corresponding to CPP in Figure 3, there are three structures of iCPP which are denoted iCPP-1, iCPP-2, and iCPP-3, dependent on the way of perturbation to the chaotic map. The structure of iCPP-1, iCPP-2, and iCPP-3 is identical to that of CPP-1, CPP-2, and CPP-3, respectively, as illustrated in Figure 3. The equations describing for iCPPs are the same those for CPPs as in Equations(14)–(20). The value of all parameters in iCPPs must be set the same as that in the corresponding CPPs to recover the original position of pixels as explained in Section 4.1. The main difference between iCPPs and CPPs is that pixels of image in iCPPs are permuted in a reverse direction in compared with that in CPPs of the encryptor, for example, from the pixel at position (M−1,N−1) backward to (0,0).

### 4.3. Chaotic Diffusion with Perturbation

**Figure 4:**
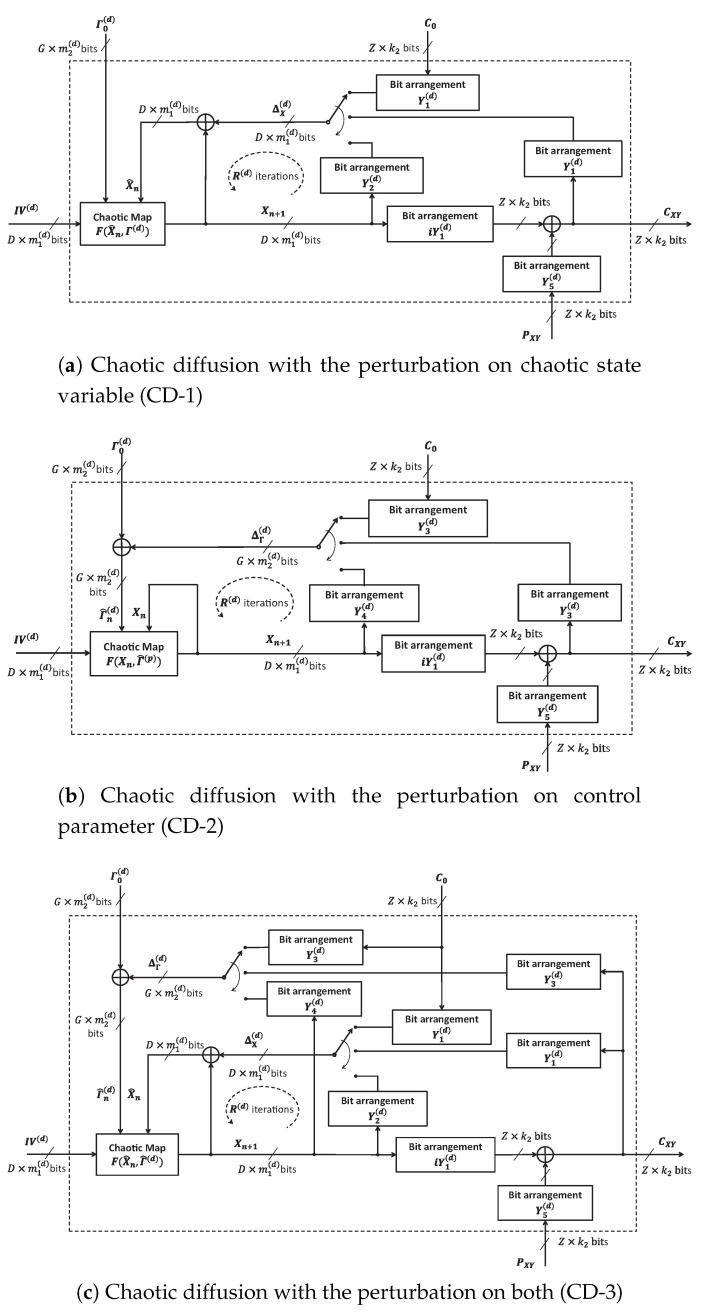
The structure of chaotic diffusions (CDs) with the perturbation.

The idea of bit-level perturbation to chaotic map as illustrated in Section 3 is again employed to propose three configurations of chaotic diffusion (CD) in Figure 4. The chaotic system is perturbed on state variables, control parameters and on both as illustrated in Figure 4a, Figure 4b, and Figure 4c, respectively. Here, the difference in these structures in compared with those of CPPs is the feedback of $C_{XY}$. Pixels are diffused sequentially. Array of bit sequences $C_{0}$ with the size of $Z\times k_{2}$ as an initial ciphertext is used for the first pixel of diffusion. $P_{XY}$ and $C_{XY}$ with the size of $Z\times k_{2}$ are arrays of bit sequences of plaintext and ciphertext, respectively. The bit arrangements in the diffusion $Y_{1}^{\left(d\right)}$, $iY_{1}^{\left(d\right)}$, $Y_{2}^{\left(d\right)}$, $Y_{3}^{\left(d\right)}$, $Y_{4}^{\left(d\right)}$, $Y_{5}^{\left(d\right)}$ and $iY_{5}^{\left(d\right)}$, are with the size of inputs and outputs as shown in Table 2. Notably, the constraint is that $Z\times k_{2}$ must be less than the number of changeable bits of state variables and control parameters in all schemes of perturbation. As a simplest application, the value of pixels is represented by a sequence of $k_{2}$ bits.

Respectively, three equations describing the diffusion as displayed in Figure 4a, Figure 4b, and Figure 4c are

(21) $$\begin{array}{c} \left\{\begin{array}{cc} {\hat{X}}_{0}=IV^{\left(d\right)}⊕\Delta_{X}^{\left(p\right)},\\ X_{n+1}=F\left({\hat{X}}_{n},\Gamma_{0}^{\left(d\right)}\right) & \;\mathrm{for}\;n=\{1\ldots R^{\left(d\right)}\}, \end{array}\right. \end{array}$$

(22) $$\begin{array}{c} \left\{\begin{array}{cc} X_{0}=IV^{\left(d\right)},\\ {\hat{\Gamma }}_{0}^{\left(p\right)}=\Gamma_{0}^{\left(p\right)}⊕\Delta_{\Gamma }^{\left(p\right)},\\ X_{n+1}=F\left(X_{n},{\hat{\Gamma }}_{n}^{\left(d\right)}\right) & \;\mathrm{for}\;n=\{1\ldots R^{\left(d\right)}\}, \end{array}\right. \end{array}$$

(23) $$\begin{array}{c} \left\{\begin{array}{cc} {\hat{X}}_{0}=IV^{\left(d\right)}⊕\Delta_{X}^{\left(p\right)},\\ {\hat{\Gamma }}_{0}^{\left(p\right)}=\Gamma_{0}^{\left(p\right)}⊕\Delta_{\Gamma }^{\left(p\right)},\\ X_{n+1}=F\left({\hat{X}}_{n},{\hat{\Gamma }}_{n}^{\left(d\right)}\right) & \;\mathrm{for}\;n=\{1\ldots R^{\left(d\right)}\}. \end{array}\right. \end{array}$$

The perturbed state variables and control parameters in Equations (21)–(23) are

(24) $$\begin{array}{c} {\hat{X}}_{n}=X_{n}⊕\Delta_{X}^{\left(d\right)},\\ {\hat{\Gamma }}_{n}^{\left(d\right)}=\Gamma_{n}^{\left(d\right)}⊕\Delta_{\Gamma }^{\left(d\right)}. \end{array}$$

There, $R^{\left(d\right)}$ is the number of iterations for each pixel in the diffusion. It is assumed that the encryption starts with the pixel at (x,y)=(0,0) toward to the last one at (x,y)=(M−1,N−1), so the arrays of bit sequences $\Delta_{X}^{\left(d\right)}$ and $\Delta_{\Gamma }^{\left(d\right)}$ in Figure 4 are

(25) $$\begin{array}{c} \Delta_{X}^{\left(d\right)}=\left\{\begin{array}{cc} Y_{1}^{\left(d\right)}\circ C_{0} & \;\mathrm{for}\;n=1\;\mathrm{and}\;(x,y)=(0,0);\\ Y_{1}^{\left(d\right)}\circ C_{XY} & \;\mathrm{for}\;n=1\;\mathrm{and}\;(x,y)\neq (0,0);\\ Y_{2}^{\left(d\right)}\circ X_{n} & \;\mathrm{for}\;2\leq n\leq R^{\left(d\right)}\;\mathrm{and}\;\forall \left(x,y\right), \end{array}\right. \end{array}$$

and

(26) $$\begin{array}{c} \Delta_{\Gamma }^{\left(d\right)}=\left\{\begin{array}{cc} Y_{3}^{\left(d\right)}\circ C_{0} & \;\mathrm{for}\;n=1\;\mathrm{and}\;(x,y)=(0,0);\\ Y_{3}^{\left(d\right)}\circ C_{XY} & \;\mathrm{for}\;n=1\;\mathrm{and}\;(x,y)\neq (0,0);\\ Y_{4}^{\left(d\right)}\circ X_{n} & \;\mathrm{for}\;2\leq n\leq R^{\left(d\right)}\;\mathrm{and}\;\forall \left(x,y\right), \end{array}\right. \end{array}$$

It is noted that $C_{XY}$ is shared between the encryptor and decryptor in the diffusion. After $R^{\left(d\right)}$ iterations, the array of bit sequences of ciphered pixels is

(27) $$C_{XY}=\left(Y_{5}^{\left(d\right)}\circ P_{XY}\right)⊕\left(iY_{1}^{\left(d\right)}\circ X_{n}\right).$$

### 4.4. Inverse Chaotic Diffusion

**Figure 5:**
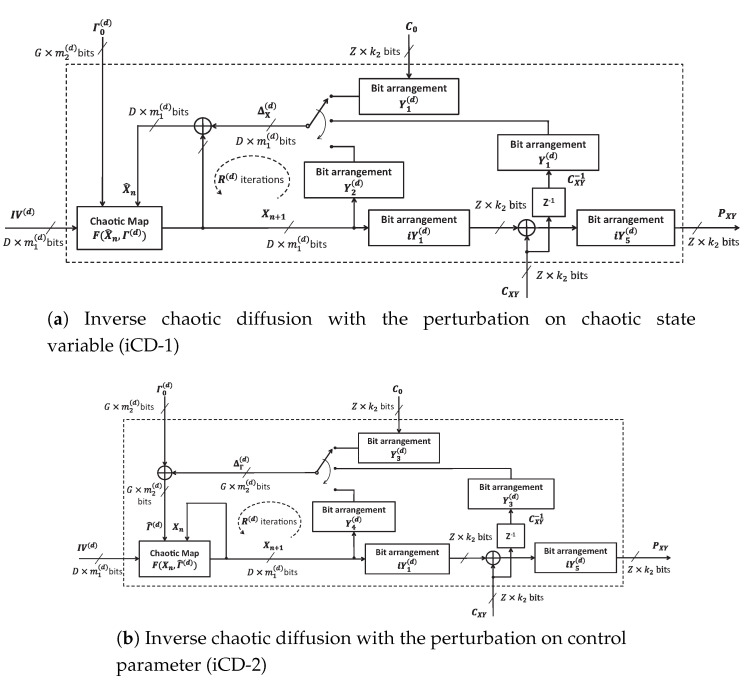

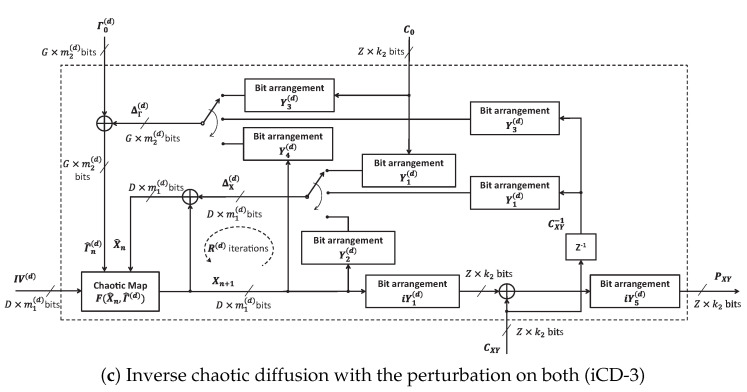
The structure of inverse CD with the perturbation.

Similarly, three configurations of Inverse Chaotic Diffusion (iCDs) in the decryptor are illustrated in Figure 5. These are almost identical to those of CDs in Figure 4, except for the additional block $Z^{-1}$ and the ciphertext $C_{XY}$ being interchanged with the plaintext $P_{XY}$ at the output. The block $Z^{-1}$ is to make the cipher data $C_{XY}$ delayed to become $C_{XY}^{-1}$ in the feedback. The equations for $\Delta_{X}^{\left(d\right)}$ and $\Delta_{\Gamma }^{\left(d\right)}$ in the decryptor are

(28) $$\begin{array}{c} \Delta_{X}^{\left(d\right)}=\left\{\begin{array}{cc} Y_{1}^{\left(d\right)}\circ C_{0} & \;\mathrm{for}\;n=1\;\mathrm{and}\;(x,y)=(0,0);\\ Y_{1}^{\left(d\right)}\circ C_{XY}^{-1} & \;\mathrm{for}\;n=1\;\mathrm{and}\;(x,y)\neq (0,0);\\ Y_{2}^{\left(d\right)}\circ X_{n} & \;\mathrm{for}\;2\leq n\leq R^{\left(d\right)}\;\mathrm{and}\;\forall \left(x,y\right), \end{array}\right. \end{array}$$

and

(29) $$\begin{array}{c} \Delta_{\Gamma }^{\left(d\right)}=\left\{\begin{array}{cc} Y_{3}^{\left(d\right)}\circ C_{0} & \;\mathrm{for}\;n=1\;\mathrm{and}\;(x,y)=(0,0);\\ Y_{3}^{\left(d\right)}\circ C_{XY}^{-1} & \;\mathrm{for}\;n=1\;\mathrm{and}\;(x,y)\neq (0,0);\\ Y_{4}^{\left(d\right)}\circ X_{n} & \;\mathrm{for}\;2\leq n\leq R^{\left(d\right)}\;\mathrm{and}\;\forall \left(x,y\right). \end{array}\right. \end{array}$$

The recovered plain pixels in the form of array of bit sequences after inverse diffusion are

(30) $$P_{XY}=iY_{5}^{\left(d\right)}\circ \left(C_{XY}⊕\left(iY_{1}^{\left(d\right)}\circ X_{n}\right)\right).$$

The value of parameters and the operation of iCD are the same as those of CD as described in Section 4.3.

### 4.5. Space of Secret Keys

It is assumed that the number of bits representing for the value of $X_{n}$ and for that of $\Gamma_{n}$ in Figure 3, Figure 4 and Figure 5 are $D\times m_{1}^{\left(d\right)}$ and $G\times m_{2}^{\left(d\right)}$, respectively. The secret keys of the proposed permutation and diffusion are the value sets of initial vectors of state variables and initial values of control parameters. It is noted that bit arrangements are considered as structural parameters rather than secret keys.

Let us define $s_{param}$ be the number of bits representing for param. Table 3 shows the number of bits encoding for values of initial vectors and control parameters for the permutation and diffusion. In fact, the number of bits representing for the secret keys is dependent on the number of perturbed bits in state variables and control parameters. Specifically, the initial value of $IV^{\left(p\right)}$, $IV^{\left(d\right)}$, $C_{0}$, $\Gamma_{0}^{\left(p\right)}$ and $\Gamma_{0}^{\left(d\right)}$) is represented in the format of fixed point and its values are varying in specific ranges. In the scheme of perturbation on control parameters, the state of some bits in the value of control parameters is fixed to ensure that chaos exhibits while that of the other bits are changeable by perturbation. Similarly in the scheme of perturbation on state variables, some selected bits of state variables are with fixed states while the others are changeable. In other words, a number of bits with fixed states do not contribute to the key space of the permutation and diffusion.

However, the number of changeable bits is as large as possible and must be larger than the number of bits encoding for the coordinate and the value of pixels in the appropriate scheme of perturbation.

### 4.6. Computational Complexity and Resource Analysis

It is emphasized that the cryptosystems working at bit level are designed with the aim to implement on hardware platforms such as Field Programmable Gate Arrays (FPGAs). Here, the computational complexity is considered in the context of using FPGAs, rather than on PC where the basic data unit is byte. In addition, the example will be given to illustrate the computational complexity.

In fact, the computational complexity and resource required for the proposed models are dependent on equations of chaotic maps and a number of bits are used for representing values of state variables and control parameters. The advantage of cryptosystems implemented on the customized hardware is that a number of bits representing for the format of fixed point can be tailored for the requirement of security and application.

The requirement of computational resource is as follows. The chaotic map requires a number of arithmetic operations and logic gates, that is, multipliers, divisors, adders, and subtractors. A number of XOR gates are used for the perturbation. A number of registers are needed to store arithmetic operands and the result. A memory space is necessary to store the plain image, and the permutation and diffusion are performed on this memory. Moreover, as it is implemented on customized hardware, all the blocks of bit arrangement in the proposed models are interconnection wires.

One of advantages in using chaotic maps for image encryption is the low computational complexity. The speed of hardware implementation is dependent mainly on the speed of arithmetic operations of chaotic map, the read/write cycles of memory during the permutation and diffusion.

For example, the Logistic map in Equation (31) is chosen for the scheme of perturbation on state variable. The hardware resource for the Logistic map is as shown in Table 4. Accordingly, it requires four registers, two multipliers, and one substractor. In addition, a number of XOR gates are necessary to implement the perturbation. In fact, it is small necessary resource to implement when it is compared with the available resource of typical FPGA devices.

## 5. Example and Simulation

It is noted that this work is to propose the design of permutation and diffusion with different schemes of perturbation, rather than a cryptosystem. In addition, due to the limit of the space, the example is mainly to demonstrate the feasibility of the approach, and only representative samples of simulation results are illustrated in case that other ones are the same. The more detail on the specific application using this approach to design a cryptosystem will be found in other papers published somewhere later.

In this example, the generic 1D Logistic map,

(31) $$x_{n+1}=ax_{n}\left(1-x_{n}\right),$$

is employed for both the permutation and diffusion.

### 5.1. Percentage of Bits Generated by Logistic Map

**Figure 6:**
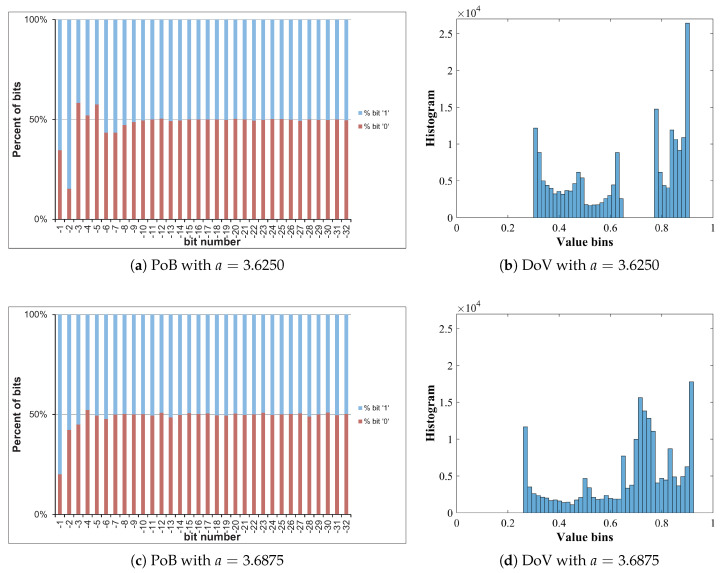

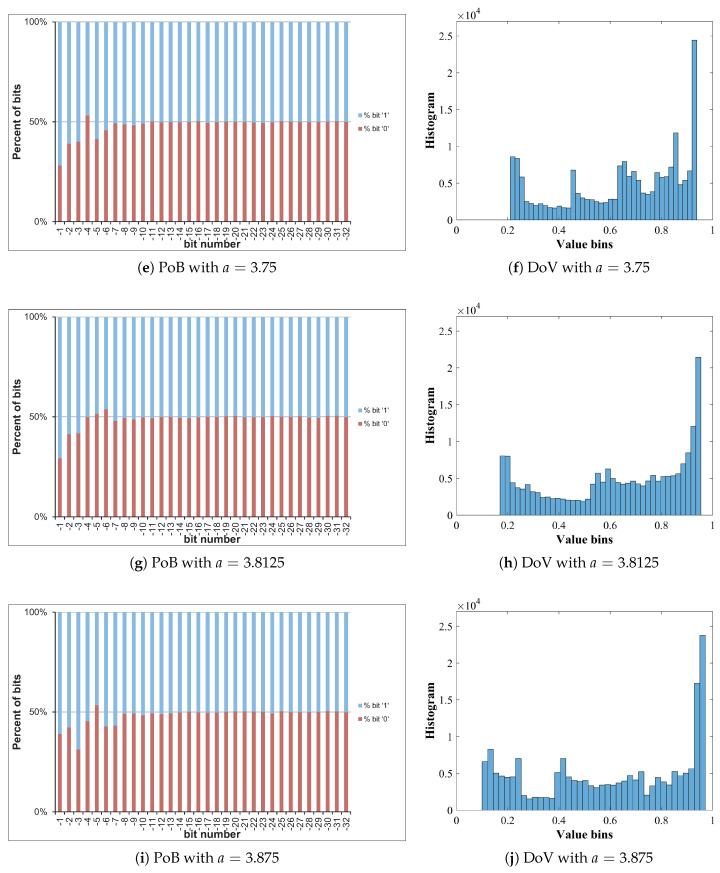

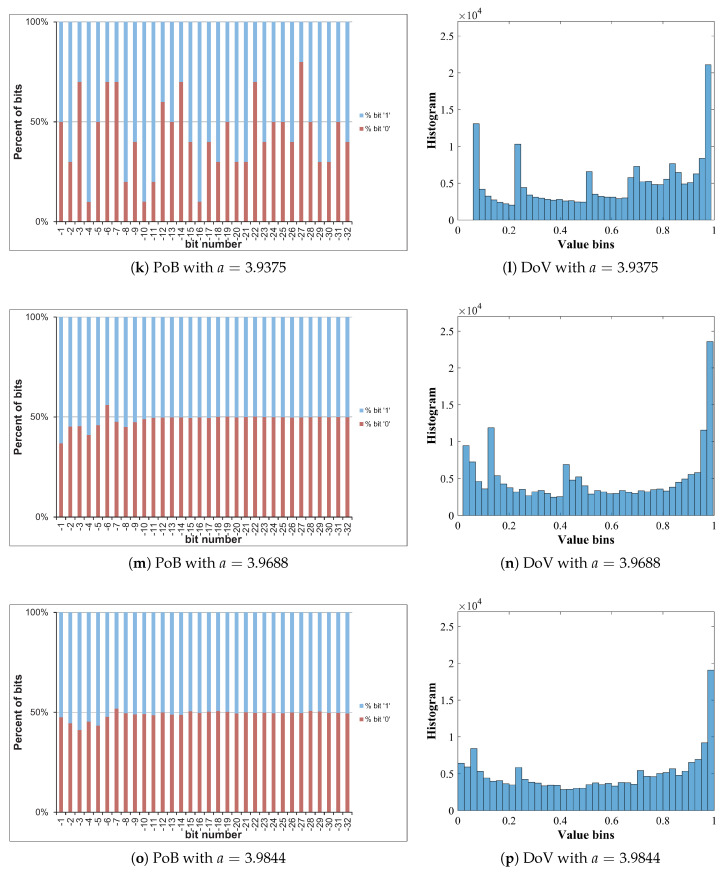

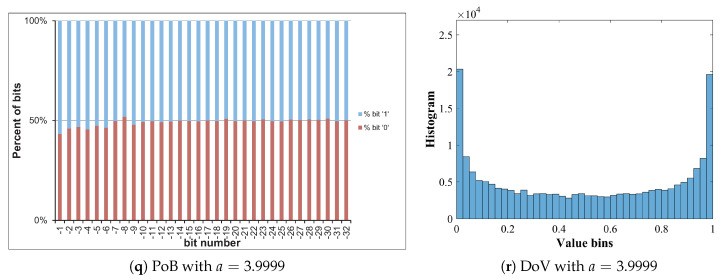
Percentage of bits and distribution of values of $x_{n}$.

The Logistic map in Equation (31) is simulated, in which values of $x_{n}$ and *a* are represented in the format of fixed point as 1.32 and 2.32, respectively. Different values of *a* are as given in Table 5. It is noted that a=3.9999 is written with 4 digits after fraction point, but in fact the exactly value is $4.0-2^{-32}$. The initial value of *x* is 0.1234567890. For each value of *a*, the Logistic map is iterated 196,608 times to produce chaotic sequences. Due to the value range of $x_{n}\in \left(0,1\right)$, so the bit $b_{0}$ representing for its integer part is always ‘0’. The percentage of bits (PoB) for bits $b_{-1}$ to $b_{-32}$ and distribution of values (DoV) of chaotic sequences generated by the Logistic map with different values of *a* are displayed in Figure 6.

It is clear from Figure 6 that the lower significant bits, that is, from bits $b_{-9}$ to $b_{-32}$, have PoBs of ‘0’ roughly equal to those of bits ‘1’ for every chosen value of *a*; whereas the PoBs of $b_{-1}$ to $b_{-8}$ are biased to either ’0’ or ’1’. Besides, DoVs of chaotic sequences are uneven for every value of *a*. The best DoV is obtained with a=3.9999 as shown in Figure 6q. Intuitively, there is a correlation between PoBs and DoVs. The better DoV is, the better PoBs of higher significant bits are obtained. In addition, PoBs for lower significant bits are independent from DoVs. It suggests that lower significant bits should be utilized for the bit-level encryption, and the value of *a* should be chosen as close to 4.0 as possible.

### 5.2. Permutation and Diffusion with Logistic Map

The 1D Logistic map in Equation (31) is employed for the permutation and diffusion, so D=1, and G=1. The notations for state variable and control parameter are $X_{n}=\left[x_{n}\right]$, $\Gamma_{n}=\left[a_{n}\right]$, $\Delta_{X}=\left[\delta_{x}\right]$ and $\Delta_{\Gamma }=\left[\delta_{a}\right]$. The superscripts (p) and (d) associate with the notations to mention the permutation and diffusion, respectively. It is noted that Logistic map exhibits chaos with 3.56995≤a≤4.0, and the value range of $x_{n}$ is (0,1).

#### 5.2.1. Chosen Value of Parameters

Values of $x_{n}$ and $a_{n}$ in the permutation and diffusion are represented in the format of fixed point as given in Table 6. The format of fixed point for the control parameters $a_{n}^{\left(p\right)}$ and $a_{n}^{\left(d\right)}$, and the state variables $x_{n}^{\left(p\right)}$ and $x_{n}^{\left(d\right)}$ is given in Table 7, in which some bits are with fixed states ‘0’ and ‘1’, and bits denoted by ‘x’ are perturbed. The bit patterns of state variables and control parameters in Table 7 indicate that the state of bits $b_{0}$ of both $x_{n}^{\left(p\right)}$ and $x_{n}^{\left(d\right)}$ is fixed at ‘0’, while that of $b_{1}$, $b_{0}$, $b_{-1}$ and $b_{-3}$ of $a_{n}^{\left(p\right)}$ and $b_{1}$, $b_{0}$, $b_{-1}$, $b_{-3}$ and $b_{-4}$ of $a_{n}^{\left(d\right)}$ is always ‘1’. The XOR operation is used as the perturbation operator. Therefore, the state of bits in perturbation amounts $\delta_{x}^{\left(p\right)}$, $\delta_{x}^{\left(d\right)}$, $\delta_{a}^{\left(p\right)}$, and $\delta_{a}^{\left(d\right)}$ must be ‘0’ at positions corresponding to bits with fixed states in $x_{n}^{\left(p\right)}$, $x_{n}^{\left(d\right)}$, $a_{n}^{\left(p\right)}$, and $a_{n}^{\left(d\right)}$, respectively.

The initial values of state variables and control parameters are chosen as in Table 8. If the perturbation is applied to, values of state variables and control parameters and amounts of perturbation will vary in the specific ranges as given in Table 9.

For simplest assumption, let us represent the coordinate and the value of pixels by a 1D sequence of bits, or Q=1 and Z=1. The 8-bit grayscale images with the size of 256×256 are encrypted, so the row and column numbers are encoded by 8 bits. In other words, $XY_{present}$ is represented by a bit sequence of $k_{1}=16$ bits as $\left(b_{15}b_{14}b_{13}b_{12}b_{11}b_{10}b_{9}b_{8}b_{7}b_{6}b_{5}b_{4}b_{3}b_{2}b_{1}b_{0}\right)$ in which the sequences $\left(b_{15}\ldots b_{8}\right)$ and $\left(b_{7}\ldots b_{0}\right)$ are encoded for values of $x_{present}$ and $y_{present}$, respectively. The value of pixels is represented by a sequence of 8 bits, that is, Z=1 and $k_{2}=8$. Therefore, The bit arrangements are chosen as in Table 10, in which the bits with fixed states in the state variables and control parameters have the position indicated by $BIT_{0}$. It is noted from the bit arrangements $Y_{2}^{\left(p\right)}$, $Y_{4}^{\left(p\right)}$, $Y_{2}^{\left(d\right)}$ and $Y_{4}^{\left(d\right)}$ in Table 10 that the bits with poor PoBs in $x_{n}$ as displayed in Figure 6 are deliberately used in the permutation and diffusion to consolidate the suggestion to utilize lower significant bits in the encryption.

In this example, the four 8-bit grayscale images [69] and two special ones with the size of 256×256 are used for the simulation, that is, Lena, Cameraman, House, and Peppers, Black and White. The simulation is carried out for the permutation and diffusion separately, and the input of the permutation and diffusion processes are the original images. The value of other parameters is chosen as: the number of iterations for each data unit in the permutation and diffusion is $R^{\left(p\right)}=10$ and $R^{\left(d\right)}=10$, respectively; and the number of permutation and diffusion rounds is $N^{\left(p\right)}=3$ and $N^{\left(d\right)}=3$.

Next, the simulation results is to show to effectiveness of the proposed schemes by means of the PoBs and DoVs of perturbed state variables and control parameters.

#### 5.2.2. Simulation Result of Permutation with Perturbation

The PoB and DoV are measured for values of state variables, control parameters as well as amounts of perturbation in the permutation and diffusion processes. It is noted that only significant samples of results are illustrated representatively to save the space.

**Figure 7:**
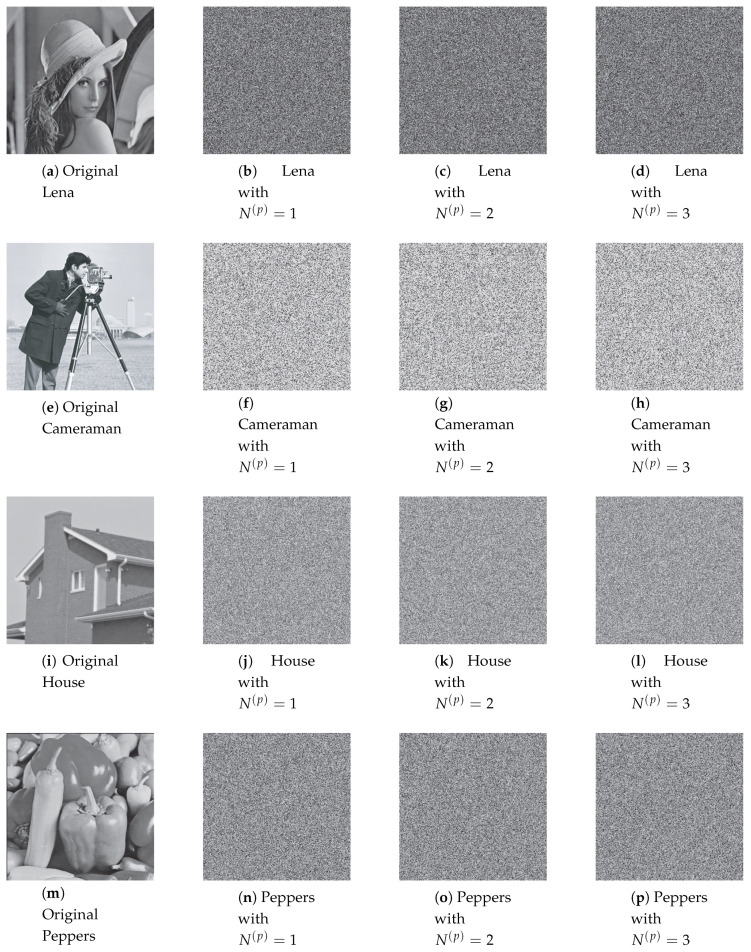
The permuted images with the perturbation on state variable.

**Figure 8:**
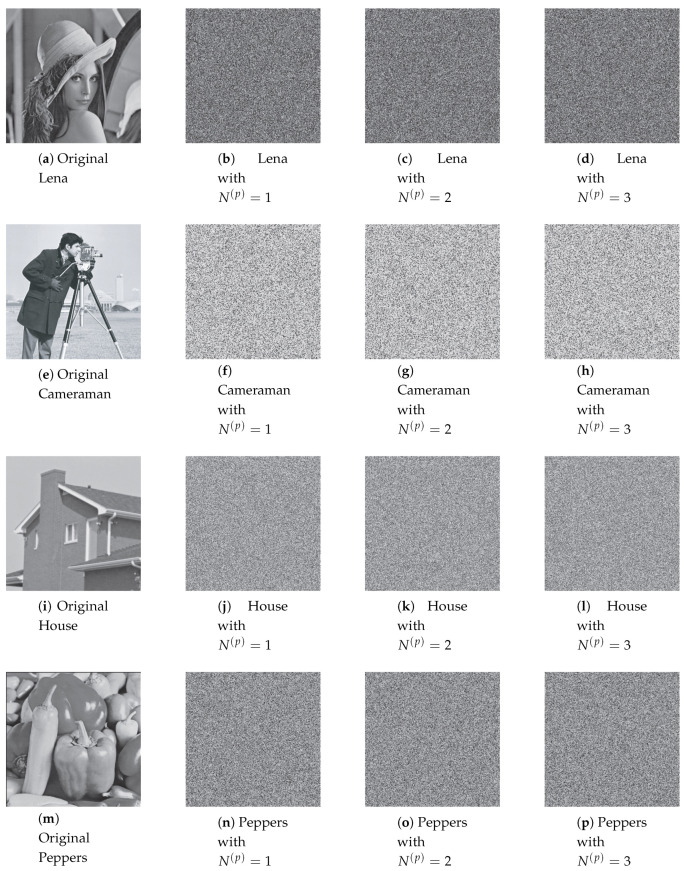
The permuted images with the perturbation on control parameter.

**Figure 9:**
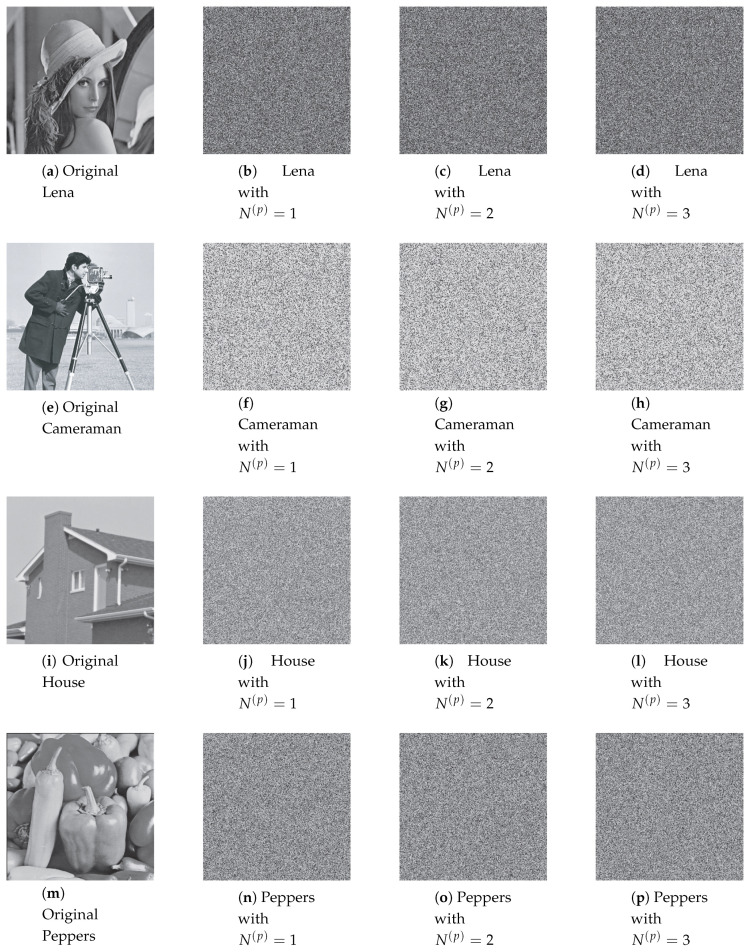
The permuted images with the perturbation on both.

Permuted images with the perturbation on the state variable, control parameter, and on both are illustrated in Figure 7, Figure 8 and Figure 9. The first column displays the original images, and the second, third and fourth columns are permuted images with different number of permutation rounds $N^{\left(p\right)}=1$, 2, and 3, respectively. It is clear that the visual structure of the original images are completely removed in the permuted images, even after the first round of permutation.

Let us analyze the DoV for state variable and control parameter, and amounts of perturbation for each scheme of perturbation. Specifically, the analysis is carried out with the chaotic sequence ${\hat{x}}_{n}^{\left(p\right)}$ and the amount of perturbation $\delta_{x}^{\left(p\right)}$ for the perturbation on state variable; with the value sequence of control parameter of ${\hat{a}}_{n}^{\left(p\right)}$ and the amount of perturbation of $\delta_{a}^{\left(p\right)}$ for the perturbation on control parameter; and with the chaotic sequence of ${\hat{x}}_{n}^{\left(p\right)}$, the value sequence of control parameter of ${\hat{a}}_{n}^{\left(p\right)}$, the amounts of perturbation of $\delta_{x}^{\left(p\right)}$ and $\delta_{a}^{\left(p\right)}$ for the perturbation on both. The PoBs for the perturbation amounts are also shown in all schemes of perturbation.

Table 7 shows the chosen pattern of bit representation for $a_{n}^{\left(p\right)}$ to explain the bias of bits in PoBs. There are some bits with the fixed state of ‘1’ and some perturbed bits with ‘x’. Due to the fixed state of bits, the value range of control parameter is broken apart to separate portions as given in Table 9, and it can be seen in Figures 11b,c and 12e,f in presenting the DoVs of control parameters and amounts of perturbation.

**Figure 10:**
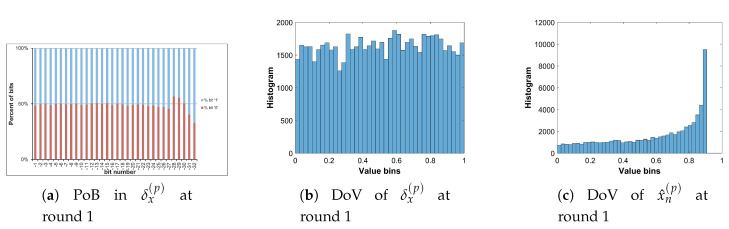
Permutation with the perturbation on state variable: PoB and DoV of amount of permutation and perturbed state variable.

**Figure 11:**
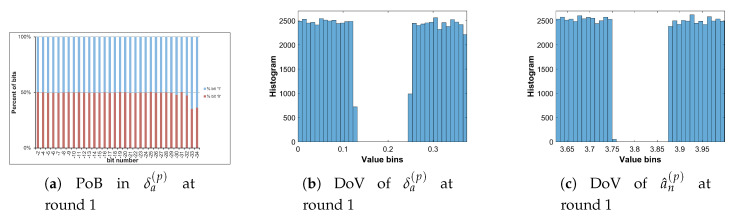
Permutation with the perturbation on control parameter: PoB and DoV of amount of permutation and perturbed control parameter.

**Figure 12:**
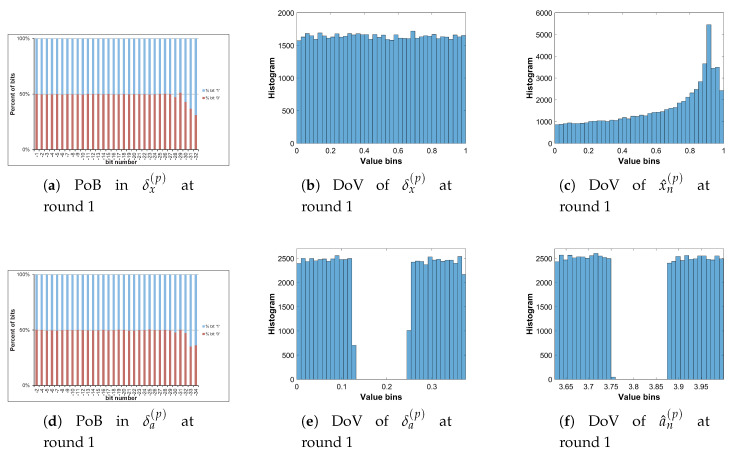
Permutation with the perturbation on both: PoB and DoV of amounts of permutation, and perturbed state variable and control parameter.

Figure 10, Figure 11 and Figure 12 illustrate for the PoBs and DoVs in the perturbation on state variable, control parameter, and on both, respectively. The PoBs are displayed in the first column, and the DoVs are in the second and third columns. Notably, the permutation process uses the coordinates of pixels (x,y) as the input. In addition, for any image with the same size, the permutation rule is the same in every permutation round for any image with the same size, or it is independent from the pixel values. Thus, for each of images, the PoBs and DoVs of the first round of permutation are shown.

It is clear from the first column of Figure 10, Figure 11 and Figure 12 that the PoBs of amounts of perturbation are even for most significant bits, and it is biased for a few lower significant bits in every scheme of perturbation. That is because the higher significant bits of $x_{n}^{\left(p\right)}$ are utilized to construct the amounts of perturbation $\delta_{x}^{\left(p\right)}$ and $\delta_{a}^{\left(p\right)}$ by the bit arrangement rules $Y_{2}^{\left(p\right)}$ and $Y_{4}^{\left(p\right)}$ in Table 10. This is agreed with the PoBs of $x_{n}$ as shown in Figure 6. In other words, the lower significant bits of $x_{n}^{\left(p\right)}$ should be employed to generate amounts of perturbation.

The DoVs of amounts of perturbation $\delta_{x}^{\left(p\right)}$ are spread over the range of (0,1) for the perturbation on state variable and on both as depicted in Figure 10b and Figure 12b. In contrast, the DoVs of perturbed state variable ${\hat{x}}_{n}^{\left(p\right)}$ cover the lower range of (0,1) for the perturbation on state variable in Figure 10c and the full range of (0,1) for the perturbation on both in Figure 12c. In addition, the DoVs of $\delta_{x}^{\left(p\right)}$ in the schemes of perturbation on state variable and on both are fairly flat while that of ${\hat{x}}_{n}^{\left(p\right)}$ is not.

As demonstrated in Figure 11 and Figure 12, the DoVs of control parameter ${\hat{a}}_{n}^{\left(p\right)}$ and its amounts of perturbation $\delta_{x}^{\left(p\right)}$ and $\delta_{a}^{\left(p\right)}$ do not cover full range of (0,1) because the bit pattern of $a_{n}^{\left(p\right)}$ is chosen as in Table 7. The bits at the positions $b_{1}$, $b_{0}$, $b_{-1}$, $b_{-3}$ are fixed at the state ‘1’, while bits at $b_{-2}$, $b_{-4}$,...$b_{-34}$ are perturbed. This makes the value range of control parameter reduced and partitioned apart. One perturbed bit in-between of two fixed bits, that is, the bit $b_{-2}$, in the fractional portion of the bit pattern of $a_{n}^{\left(p\right)}$ makes the value ranges of $\delta_{a}^{\left(p\right)}$ and ${\hat{a}}_{n}^{\left(p\right)}$ divided into two separate portions as shown Figure 11 and Figure 12. That is agreed with the portions of value ranges given in Table 9. In general, there are $2^{n_{b}}$ separate portions of value ranges for $n_{b}$ perturbed bits in-between fixed bits.

#### 5.2.3. Simulation Result of Diffusion with Perturbation

**Figure 13:**
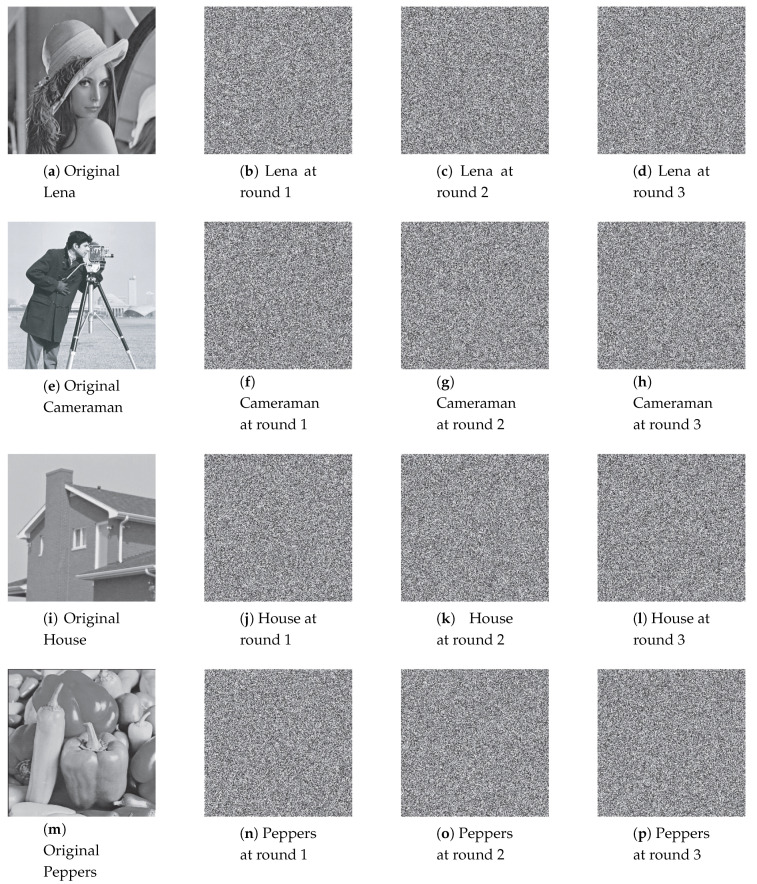
The diffused images with the perturbation on state variable.

**Figure 14:**
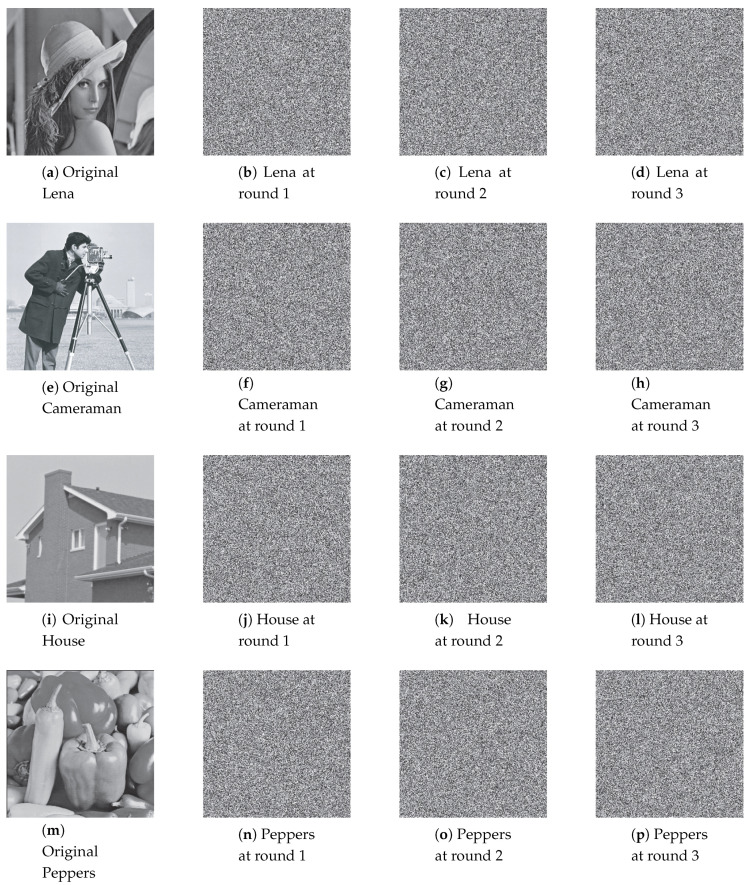
The diffused images with the perturbation on control parameter.

**Figure 15:**
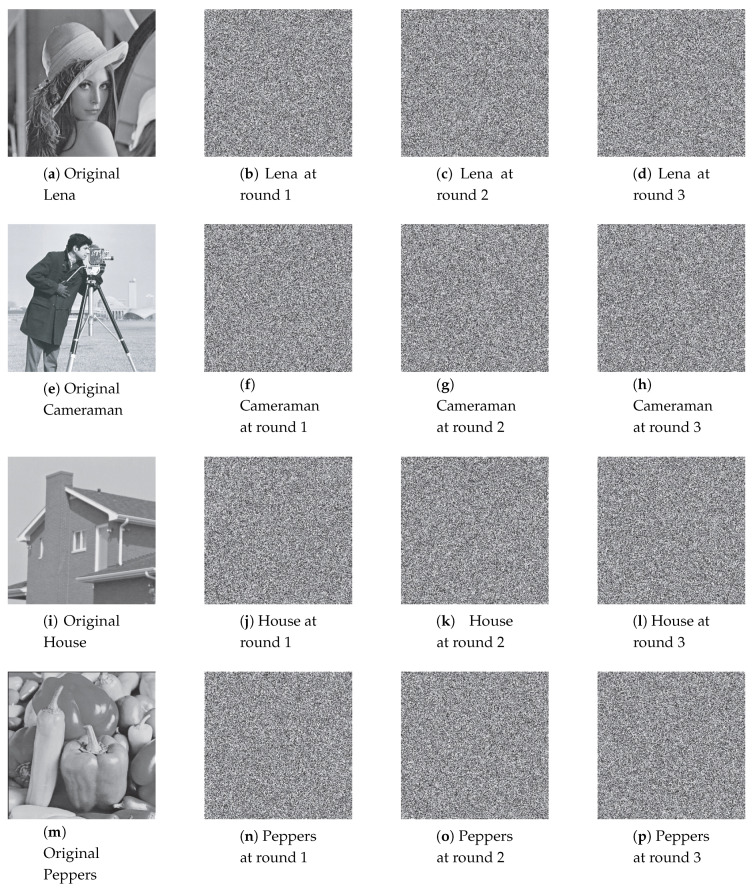
The diffused images with the perturbation on both.

Figure 13, Figure 14 and Figure 15 illustrate the original images and its corresponding diffused ones in the second, third, and fourth columns with different number of diffusion rounds, that is, $N^{\left(d\right)}=1$, 2, and 3. Note that each pixel is iterated ten times ($R^{\left(d\right)}=10$). It is clear that the visual structure of the original images is completely destroyed in the diffused images, even after the first round of diffusion.

**Figure 16:**
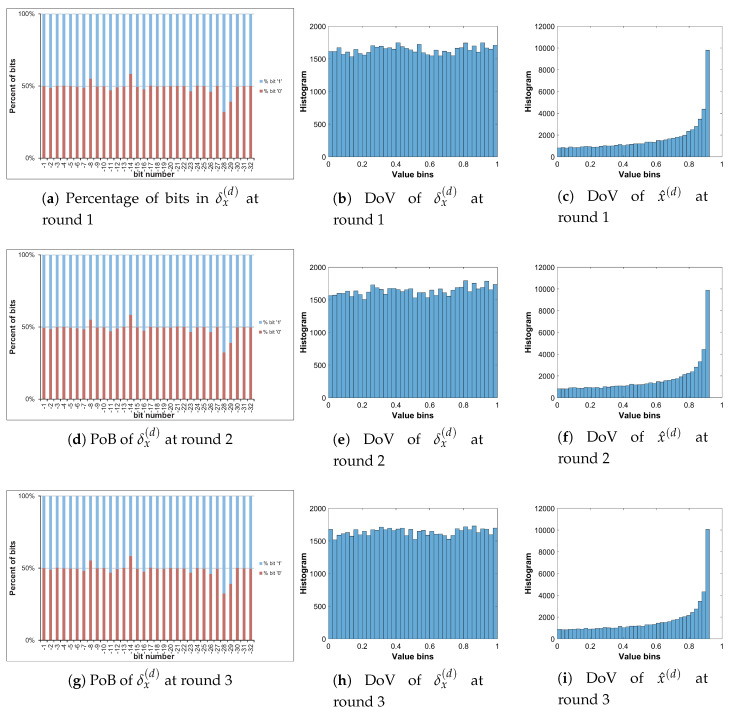
Diffused Cameraman: PoBs and DoVs with the perturbation on state variable.

**Figure 17:**
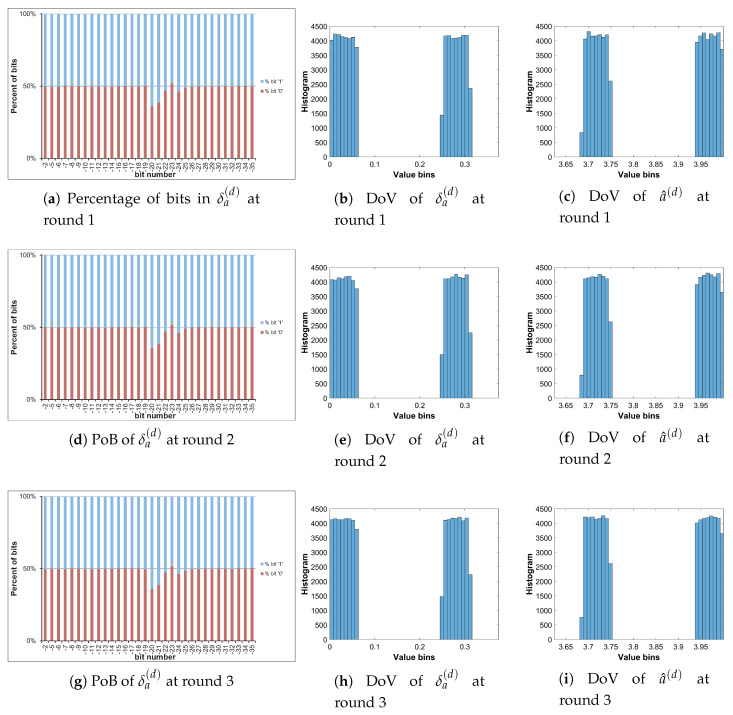
Diffused Cameraman: PoBs and DoVs with the perturbation on control parameter.

**Figure 18:**
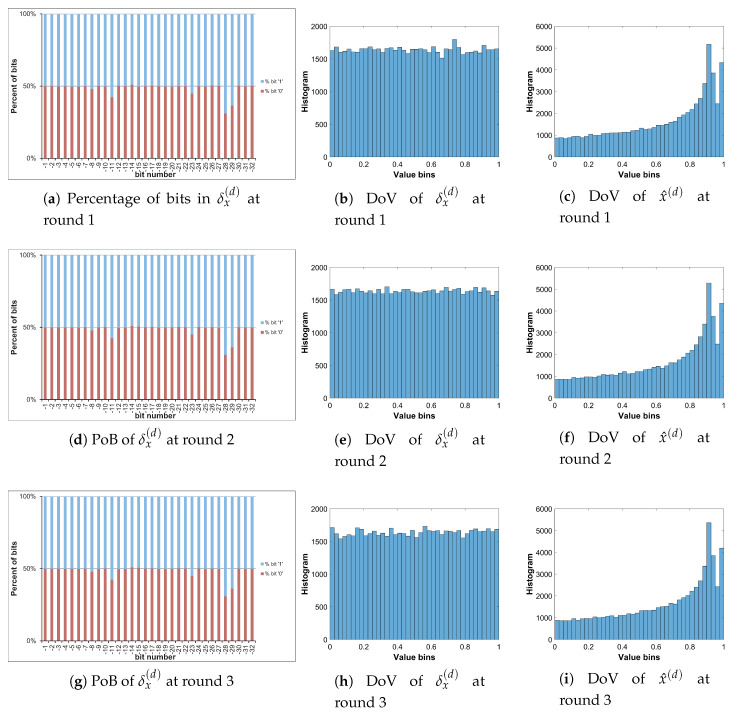
Diffused Cameraman: PoBs and DoVs of state variable with the perturbation on both.

**Figure 19:**
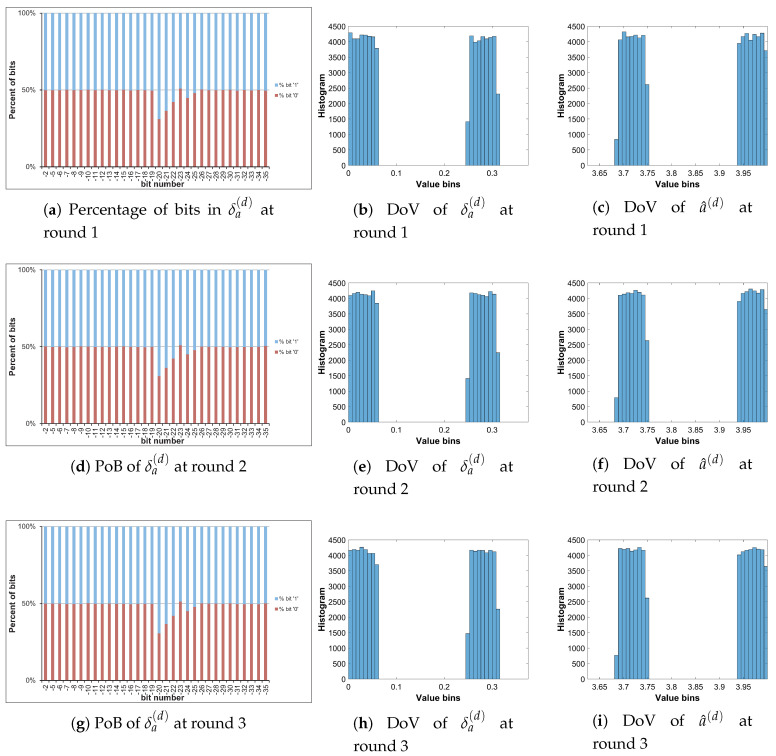
Diffused Cameraman: PoBs and DoVs of control parameter with the perturbation on both.

To save the space, the PoBs and DoVs in the diffusion of only Cameraman image are illustrated in Figure 16, Figure 17, Figure 18 and Figure 19. The result shows almost the same to those in the permutation as described above.

The PoBs in the first column shows the bias to bit ‘1’ at the bit positions $b_{-28}$ and $b_{-29}$ of $\delta_{x}^{\left(d\right)}$ in Figure 16 and Figure 18, and at $b_{-20}$, $b_{-21}$, $b_{-22}$, $b_{-24}$ and $b_{-25}$ of $\delta_{a}^{\left(d\right)}$ in Figure 17 and Figure 19. The bias also occurs to bit ‘0’ at the bit positions $b_{-8}$ and $b_{-14}$ of $\delta_{x}^{\left(d\right)}$ in Figure 16. As described by $Y_{2}^{\left(d\right)}$ and $Y_{4}^{\left(d\right)}$ in Table 10, the bias is caused by higher significant bits of ${\hat{x}}_{n}^{\left(d\right)}$ employed to construct the amounts of perturbation $\delta_{x}^{\left(d\right)}$ and $\delta_{a}^{\left(d\right)}$. This is also agreed with the PoBs of $x_{n}$ as shown in Figure 6. Similar to above permutation, the lower significant bits of $x_{n}^{\left(d\right)}$ should be chosen to generate amounts of perturbation in the diffusion.

#### 5.2.4. Space of Secret Keys

The secret keys in the proposed permutation and diffusion are the initial values of state variables and control parameters. In fact, values of state variables and control parameters are changed during perturbation. The Logistic map in chaotic behavior requires the control parameter and the state variable varying in defined ranges. That is, the integer portions of values of state variables and control parameters must be ‘0’ and ‘11’, respectively. In addition, there are some bits in the fractional portions of control parameters must be kept constant at the state of ‘1’, for example, bit $b_{-1}$, to ensure that the value range of control parameters in (3.56995,4.0). Therefore, the constraints make the initial values of state variables and parameters contribute the number of bits to the secret keys less than its definition.

According to the adopted values of parameters for the permutation and diffusion in this example, the number of bits represents for the secret keys is dependent on not only that of perturbed bits, but also the constraints in the value ranges of state variables and control parameters of chaotic map. Table 11 shows the number of bits for the secret keys of permutation and diffusion in different schemes of perturbation. The values of control parameters, $a_{0}^{\left(p\right)}$ and $a_{0}^{\left(d\right)}$, in the scheme of perturbation on the state variables (CPP-1 and CD-1) are fixed, so those contribute 33 and 34 bits to the secret keys, respectively, while other initial values provide 32 bits as defined by the bit patterns in Table 7. In other words, the number of bits in the secret keys can be at least 64 and 72 for the permutation and diffusion, respectively.

It is assumed that the cryptosystem consists of the permutation and diffusion with the perturbation of Logistic map as described above. Therefore, the secret key of a cryptosystem is at least 136 bits in length. That is long enough to resist from the brute force attack running on nowadays computers.

#### 5.2.5. Statistical Analyses

Here, some appropriate statistical analyses related to the content of images are carried out for the exemplar structures of permutation and diffusion. That is, the histogram, information entropy, correlation coefficients, sensitivity of secret keys, measurement of quality, and chosen-plaintext attack as well as chosen-ciphertext one are computed for this example. In the presentation of results in the tables, bad results and the best one are in italic and in bold, respectively.

It is noted that it is unbiased to compare the statistical measures for the permutation and diffusion processes in this work with those obtained by a whole cryptosystem. The simulation result is compared with that in recent works to show the advantages. However, the statistical measures for each of the permutation and diffusion show the separate contribution, if these are employed to construct a cryptosystem.

1. *Histogram analysis*
  Histogram reflects the distribution of pixel values of an image. Histogram analysis of an image is considered by means of statistical histogram. The $\chi^{2}$ is measured for statistical histogram. It is defined by
  (32) $$\chi^{2}=\sum_{i=0}^{K-1}\frac{{\left(O_{i}-E_{i}\right)}^{2}}{E_{i}},$$
  where *K* is the number of grey level (K=256 for 8-bit grayscale images), and $O_{i}$ and $E_{i}$ are respectively observed and expected occurrence frequencies of gray level *i*, with 0≤i≤K−1. Expected occurrence frequencies of 8-bit grayscale images is $E_{i}=\frac{M\times N}{K}$; *M* and *N* are the number of rows and columns of images. The unilateral hypothesis test is to consider the significance of the histogram conforming a uniform distribution. The hypothesis test is accepted (or the histogram is uniformly distributed) if $\chi^{2}\leq \chi_{\alpha }^{2}\left(K-1\right)$. In this example, the significance level α=0.05 is considered and $\chi_{0.05}^{2}\left(255\right)=293.247$.
  It is noted that the analysis of histogram is only applied to the diffusion. Four original images and two special images (Black and White images) are employed in the simulation for the histogram analysis. Table 12 shows values of $\chi^{2}$ which are computed for original and diffused images for different rounds of diffusion. The $\chi^{2}$ values of original images are quite large in compared with those of diffused ones. It means that the histograms of original images have clear structures. Specifically, the $\chi^{2}$ values of most diffused images are less then $\chi_{0.05}^{2}\left(255\right)$ after the first round of diffusion, or the histograms of diffused images have uniform distributions. The diffused images of Black and White have uniform histograms from the third round of iteration. However, histogram structures still exist in the first-round diffused Black and White images. It seems that there is not much difference in $\chi^{2}$-test result in different schemes of perturbation. The test results show that the diffusion process provides the histogram statistics equivalent to those produced by a whole cryptosystem for example, Reference [58].
2. *Information entropy*
  The information entropy IE(V) is used for measuring the probability of appearance of symbol $v_{i}$ in the message source *V* [70]. Here, the message source is the encrypted images and symbols are pixels. Calculation of IE(V) for an image is
  (33) $$IE\left(V\right)=\sum_{i=0}^{2^{k_{2}}-1}p\left(v_{i}\right)log_{2}\frac{1}{p(v_{i})},$$
  where $p(v_{i})$ is the probability in finding pixels with value of $v_{i}$ in an image. IE(V) is in bit. In the case of 8-bit grayscale image, the maximum of $IE(v_{i})$ is 8 as the ideal value. Here, the entropy is only considered for diffused images only, because the permutation does not change values of pixels. Under a cryptographic point of view, the better the statistical property in a diffused image, the closer the value of IE(V) to the ideal one.
  Table 13 presents the information entropy of diffused images obtained by different schemes of perturbation. For four test images, the entropy of original images is much less than the ideal one, while that of most diffused images is very close to the ideal one, that is, larger than 7.99 regardless to the scheme of perturbation and the number of diffusion rounds as well. However, the diffused images of Black and White have low entropy at the first round of diffusion and it increases to the ideal one at the second and third round of diffusion. The result shows that the information entropy of diffused images are equivalent to that in most previous works, for example, References [27,29,58,62].
3. *Correlation coefficient*
  The correlation coefficient (CC) among adjacent pixels reflects one of visual properties of images, and it is high in natural images. the CCs in three directions, that is, horizontal, vertical and diagonal adjacency, are measured for a specific pixel. Thus, it is expected that CCs are close to zero in encrypted images.
  Here, the CCs are considered for both permuted and diffused images, and are computed on the full range of images. Table 14, Table 15, Table 16, Table 17, Table 18 and Table 19 show the CCs of permuted, original and diffused images for four test images. Due to special content, the CCs are computed for only diffused Black and White images. The CCs are around or larger than 0.9 for four test images, and are infinity for Black and White images. Those of transformed images are relatively close to zero, and seem to be independent from chosen scheme of perturbation and from the number of rounds. In other words, the visual structure are removed in transformed images. The result of correlation coefficients is also comparable to that given in recent reports, for example, References [27,29,58,62].
4. *Sensitivity of secret keys*
  The sensitivity of secret key is considered by means of ciphertext difference rate (CDR) as proposed in Reference [71]. The CDR is computed by
  (34) $$Cdr=\frac{Diff\left(C,C_{1}\right)+Diff\left(C,C_{2}\right)}{2M\times N}\times 100\%,$$
  where *M* and *N* are the size of images; *C* is the ciphertext using the secret key *K*; $C_{1}$ and $C_{2}$ are ciphertexts using the secret keys K+ΔK and K−ΔK, respectively; the function Diff(A,B) returns the difference in the number of pixels between images *A* and *B*. The function Diff(.) is
  (35) $$Diff\left(A,B\right)=\sum_{x=1}^{M}\sum_{x=1}^{N}Difp\left(A\left(x,y\right),B\left(x,y\right)\right),$$
  where Difp(.) is
  (36) $$Difp\left(A\left(x,y\right),B\left(x,y\right)\right)=\left\{\begin{array}{cc} 1, & \mathrm{for}\;A(x,y)\neq B(x,y),\\ 0, & \mathrm{for}\;A(x,y)=B(x,y). \end{array}\right.$$
  It is clear that the value difference in pairs of pixels is considered for the CDR. Thus, this can be used for analyzing the sensitivity of secret keys in both the permutation and diffusion for four test images, and only in the diffusion for two special images, Black and White.
  Here, the secret keys are initial values of ($IV^{\left(p\right)}$, $a_{0}^{\left(p\right)}$) for the permutation, and ($IV^{\left(d\right)}$, $a_{0}^{\left(d\right)}$) for the diffusion. Thus, the sensitivity will be considered for each of components of the secret key, and $\Delta K_{name}^{\left(Scheme_{i}\right)}$ denotes the difference in the component name of the scheme $Scheme_{i}$. In order to demonstrate the effectiveness, only the value of a single component of the secret key is added to and subtracted from the tolerance $\Delta K_{name}^{\left(Scheme_{i}\right)}$ to produce $C_{1}$ and $C_{2}$ while the other values are as previously chosen for the above simulation. The smallest value is made by the lowest significant bit for $\Delta K_{name}^{\left(Scheme_{i}\right)}$ in different schemes of perturbation as shown in Table 20.
  The simulation is carried out with four test images and two special ones, Black and White and the results are shown in Table 21, Table 22, Table 23, Table 24, Table 25 and Table 26 for the example that Cdr_IV, Cdr_a and $Cdr\_C_{0}$ are the ciphertext difference rates with a tolerance in three initial values, IV, *a*, and $C_{0}$, respectively. Overall, Cdr_IV, Cdr_a and $Cdr\_C_{0}$ are very close to unity with smallest tolerances in each component of the secret keys for any round of diffusion and for every scheme of perturbation. Specifically, for all images, the diffusion produces very good sensitivity to secret key with Cdr larger than 0.994 for every scheme of perturbation. However, for four test images, for the perturbation on state variable, the sensitivity to Cdr_IV is worse at the first round of permutation than that in larger number of permutation rounds. For every scheme of perturbation, sensitivity to Cdr_a is worse at the first round of permutation than that in larger number of permutation rounds. The result is obtained with the bit arrangements as given in Table 10, and it can be improved if higher significant bits of $x_{n}^{\left(p\right)}$ and $x_{n}^{\left(d\right)}$ are avoided to generate amounts of perturbation. Here, the result of CDRs is comparable to that in Reference [27].
  In addition, the sensitivity to the secret keys can be considered by means of number of pixels change rate (NPCR) and unified average changing intensity (UACI) [72,73]. These are as
  (37) $$NPCR=\frac{\sum_{x,y}D\left(x,y\right)}{M\times N}\times 100\%,$$
  and
  (38) $$UACI=\frac{1}{N^{2}}\left[\sum_{x,y}\frac{c_{1}\left(x,y\right)-C_{2}\left(x,y\right)|}{255}\right]\times 100\%,$$
  where D(x,y)=1 if $C_{1}\left(x,y\right)=C_{2}\left(x,y\right)$, and D(x,y)=0 if $C_{1}\left(x,y\right)\neq C_{2}\left(x,y\right)$; $C_{1}$ and $C_{2}$ are described in Equation (34). The tolerance in the secret keys is as shown in Table 20. The simulation for six test images with the permutation and diffusion using the secret keys with and without the tolerance. The resultant images are used to compute for NPCR and UACI. It is noted that only these are inappropriate for permuted images of Black and White.
  Table 27, Table 28, Table 29, Table 30, Table 31 and Table 32 demonstrated NPCR and UACI for the permuted and diffused images with various schemes of perturbation. Generally, for every scheme of perturbation and for test images except two special content ones (Black and White), NPCR of permutation is increased with the increase of number of rounds, and it is lower than that of diffusion for every component of secret keys. NPCR of diffusion is saturated and fluctuated in the range of 99.4% to 99.7% regardless of number of diffusion rounds, schemes of perturbation, and components of secret keys.
  Similarity, for test images except for Black and White and for every scheme of permutation, UACI of permutation is increased with the increase of number of permutation rounds, and it is lower than that of diffusion. UACI of diffusion is fluctuated within the range of 31.7% to 34.7% for every scheme of perturbation and for every component of secret keys. However, UACI of permutation is different for different test images, and it is better sensitivity to $\Delta_{IV}$ than to $\Delta_{a}$.
  The values of NPCR and UACI of diffusion in this work are equivalent to those of encrypted images by a whole cryptosystem in most of previous works, for example, References [27,29,58,62].
5. *Measurement of permutation and diffusion quality*
  Here, the quality of permutation and diffusion of three schemes of perturbation in the example is measured using the test images by the Mean-Squared-Error (MSE) and Peak Signal-to-Noise Ratio (PSNR). Those are performed to compare the plain images *P* and permuted ones *C* as
  (39) $$MSE=\frac{1}{M\times N}\sum_{x=1}^{M}\sum_{y=1}^{N}{\left|P\left(x,y\right)-C\left(x,y\right)\right|}^{2},$$
  where, P(x,y) and C(x,y) are values of pixels at (x,y) in *P* and *C*, respectively, and
  (40) $$PSNR=20\times log_{10}\left\{\frac{255}{sqrt(MSE)}\right\}.$$
  The larger value of MSE is, the higher quality of permutation is obtained. In contrast, the value of PSNR is expected as small as possible. Table 33 shows the quality of permutation by means of MSE and PSNR for four images excepted for Black and White. Values of MSE for the images are large, and those of PSNR are small correspondingly. It means that most pixels of the plain image *P* are with values different from those in permuted one *C*, or high quality of permutation is obtained. However, the result shows that values of MSE and PSNR are only unequal for different plain images, but independent from the schemes of perturbation and the number of permutation rounds.
  Besides, both MSE and PSNR are also used for measuring the quality of diffusion. In addition, the sensitivity to the plain images and diffused ones is characterized the quality of diffusion by means of NPCR and UACI. These are considered as follows. A pair of plain images, *P* and $P_{1}$ are diffused, in which $P_{1}$ is a modified version of *P* with a small change by the state of the least significant bit (LSB). The corresponding pair of diffused images *C* and $C_{1}$ are obtained for analyzing the sensitivity to the plaintext. Similarly, the image $C^{\prime }$ is achieved by modifying the diffused image *C*, and then inversely diffused to obtain the recovered plain image $P^{\prime }$. Here, the diffusion and inverse diffusion processes are carried out on sequential pixels, therefore, the modification is made to the first pixels of *P* and $C^{\prime }$. Here, the NPCR and UACI are given in Equations (37) and (38), and computed on the pairs of (*C*, $C_{1}$) and (*P*, $P^{\prime }$) for analyzing the sensitivity to plaintext and ciphertext.
  Table 34 and Table 35 display the MSE, PSNR, NPCR and UACI calculated for six pairs of test images, that is, (*C*, $C_{1}$) and (*P*, $P^{\prime }$), to measure the quality of diffusion and inverse diffusion. Clearly, large values of MSE, NPCR and UACI, and small values of PSNR are obtained. It means that with small tolerances in *P* and *C* generate huge difference in $C_{1}$ and $P^{\prime }$, respectively; or high quality of diffusion is achieved. Overall, all of measures are independent from the schemes of perturbation and the number of diffusion rounds.
  In detail, values of MSE and PSNR of diffusion in Table 34 are dependent on the content of plain images, while those of NPCR and UACI are not. Values of MSE and PSNR of Cameraman, Black and White images in the diffusion is better than those of Lena, House and Peppers images.
  As given in Table 35 for the inverse diffusion, values of not only MSE, PSNR, but also UACI are dependent on the content of plain images, and those measures of Cameraman, Black and White images are larger than those of Lena, House and Peppers. Values of UACI of Black and White images are extremely good, while those of House are worse.
  The quality for each of permutation and diffusion processes in this example is better than those in recent works, for example, Reference [74,75].
6. *Chosen-plaintext and chosen-ciphertext attacks*
  In this work, the permutation and diffusion processes are considered separately. According to the structure of perturbation as given in Section 4.1 and the figures therein, the permuted image in does not depends on the content of plain image. In other words, the permutation algorithms can not resist again chosen-plaintext and chosen-ciphertext attacks. However, the permutation process usually combines with a diffusion one in construction of cryptosystem.
  Here, the diffusion algorithms as described in in Section 4.3 and Section 4.4 have image-content sensitivity. The value of pixels are perturbed on the state variables and control parameters of chaotic map. This is similar to the case of authentication as given in References [57,76,77], where the hashed keys with limited lengths (e.g., 256 bits) are computed using the content of image. However, the better advantage in the proposed models in compared with previous works is that the the diffused image is dependent on every value of pixels, or it means that the length of hashed keys is equal to that of image in bits, that is, $M\times N\times k_{2}$ bits. Consequently, the diffusion algorithms strongly resist from the types of chosen-plaintext and chosen-ciphertext attacks.
  The simulation result in this example in Table 34 and Table 35 shows the image-content sensitivity by means of MSE, PSNR, NPCR and UACI as the evidence of the image-content sensitivity and resistance from chosen-plaintext and chosen-ciphertext attacks.

## 6. Concluding Remarks

The present work has proposed the structural models of image permutation and diffusion based on perturbed digital chaos. Dynamics of chaotic map is nonstationary during encryption. This introduces a class of chaotic ciphers utilizing the perturbation. To demonstrate the feasibility of the proposed models, the example employed the simplest chaotic map, that is, Logistic map. The simulation results of permutation and diffusion have been analyzed separately. Overall, the best result is obtained in the case of perturbation on both state variable and control parameter. The results are comparable to those reported in recent works, for example, References [27,55] and References [27,29,58,62]. There are some remarks in the proposed models of permutation and diffusion with the perturbed chaos.

Due to the dependency of image content, it should be ensured in any specific design that dynamics of chaos has good statistical properties and the cryptographic performance is obtained for special image contents. In fact, any chaotic map can be employed for the proposed models. A requirement for implementation is that the total number of perturbed bits in state variables or control parameters in a specific scheme of perturbation must be equal or larger than that representing for the coordinates and values of pixels. In addition, the key space of the proposed schemes is dependent on the number of perturbed bits. This can be expanded with the increase in the number of bits represented for state variables and control parameters in appropriate scheme of perturbation. It also means that the period of dynamics is lengthened. Besides, bits with fixed states in the value of state variables and control parameters will make value ranges of state variables and control parameters valid in separate intervals. The number of bits representing for chaotic variables and control parameters should be chosen to keep balanced between the expected size of key space and the resource available in the implementation platform.

Moreover, the structure of permutation is almost similar to that of diffusion in the same scheme of perturbation. The main difference in the structures is the way that the coordinate and the value of pixels are perturbed on state variables and control parameters, and in their recovery processes from the state variables. In the proposed structures, the rule of perturbation by means of controlling the switching is defined by Equations (19) and (18) for the permutation and by Equations (26) and (25) for diffusion. This can be changed to have better security performance. For specific sizes of images, the modulo operation can be used to figure out new coordinate of pixels in the case that the size of images along any axis is unequal to $2^{n}$; *n* is an integer.

Lastly, the required resource for hardware implementation is quite low in compared with typical FPGAs. In addition, there is no operation of comparison in the hardware, thus these models can have high speed operation. Further speed can be improved by combining more than one coordinate or value of pixels perturbing on chaotic dynamics at a time. This is allowed in the case the number of perturbed bits is large enough to attain that of bits of coordinates or values of pixels. The models can be simply realized in hardware with the use of multipliers, adders, XOR gates and switches. Hardware design will be implemented on FPGAs as the future work of the proposed models.

## Figures and Tables

**Table 1:** Bit arrangement.

Bit Arrangements	Size of Inputs	Size of Outputs
$Y_{1}^{\left(p\right)}$	$Q\times k_{1}$	$D\times m_{1}^{\left(p\right)}$
$Y_{3}^{\left(p\right)}$		$G\times m_{2}^{\left(p\right)}$
$iY_{1}^{\left(p\right)}$	$D\times m_{1}^{\left(p\right)}$	$Q\times k_{1}$
$Y_{2}^{\left(p\right)}$		$D\times m_{1}^{\left(p\right)}$
$Y_{4}^{\left(p\right)}$		$G\times m_{2}^{\left(p\right)}$

**Table 2:** Bit arrangement.

Bit Arrangements	Size of Inputs	Size of Outputs
$Y_{1}^{\left(d\right)}$	$Z\times k_{1}$	$D\times m_{1}^{\left(d\right)}$
$Y_{3}^{\left(d\right)}$		$G\times m_{2}^{\left(d\right)}$
$iY_{1}^{\left(d\right)}$	$D\times m_{1}^{\left(d\right)}$	$Q\times k_{1}$
$Y_{2}^{\left(d\right)}$		$D\times m_{1}^{\left(d\right)}$
$Y_{4}^{\left(d\right)}$		$G\times m_{2}^{\left(d\right)}$
$Y_{5}^{\left(d\right)}$	$Z\times k_{2}$	$Z\times k_{2}$
$iY_{5}^{\left(d\right)}$		

**Table 3:** The maximum number of bits representing for the initial values.

Parameter	Maximum Number of Bits
$IV^{\left(p\right)}$	$s_{IV^{\left(p\right)}}$
$IV^{\left(d\right)}$	$s_{IV^{\left(d\right)}}$
$\Gamma_{0}^{\left(p\right)}$	$s_{\Gamma^{\left(p\right)}}$
$\Gamma_{0}^{\left(d\right)}$	$s_{\Gamma^{\left(d\right)}}$
$C_{0}$	$s_{k_{2}}$

**Table 4:** Hardware components to implement the Logistic map.

Term	Register (Buffer)	Multiplier	Substractor
$x_{n}$	✓		
*a*	✓		
$T_{1}=a*x_{n}$	✓	✓	
$T_{2}=\left(1-x_{n}\right)$			✓
$T_{1}*T_{2}$	✓	✓	

**Table 5:** Chosen values of *a* for the percentage of bits (PoB) and distribution of values (DoV) analysis.

Chosen Values of *a*	Bit Representation in the Format of 2.32
3.6250	11.10100000000000000000000000000000
3.6875	11.10110000000000000000000000000000
3.7500	11.11000000000000000000000000000000
3.8125	11.11010000000000000000000000000000
3.8750	11.11100000000000000000000000000000
3.9375	11.11110000000000000000000000000000
3.9688	11.11111000000000000000000000000000
3.9844	11.11111100000000000000000000000000
3.9999	11.11111111111111111111111111111111

**Table 6:** The number of bits representing for the value of state variables and control parameters of Logistic maps, and for the coordinate and the value of pixels in the permutation and diffusion.

Parameter	No. of Bits	The Format
$m_{1}^{\left(p\right)}$	33	1.32
$m_{2}^{\left(p\right)}$	36	2.34
$m_{1}^{\left(d\right)}$	33	1.32
$m_{2}^{\left(d\right)}$	37	2.35
$k_{1}$	16	16.0
$k_{2}$	8	8.0

**Table 7:** Bit patterns of state variables and control parameters.

State Variables & Parameters	Patterns of Bit Representation
$a_{n}^{\left(p\right)}$	11.1 × 1xxxxxxxxxxxxxxxxxxxxxxxxxxxxxxx
$a_{n}^{\left(d\right)}$	11.1 × 11xxxxxxxxxxxxxxxxxxxxxxxxxxxxxxx
$x_{n}^{\left(p\right)}$	0.xxxxxxxxxxxxxxxxxxxxxxxxxxxxxxxx
$x_{n}^{\left(d\right)}$	0.xxxxxxxxxxxxxxxxxxxxxxxxxxxxxxxx

**Table 8:** Initial values of cryptosystem’s parameters.

Parameter	Initial and Adopted Values
$a_{0}^{\left(p\right)}$	3.6250
$a_{0}^{\left(d\right)}$	3.68750
$IV^{\left(p\right)}$	0.0123456789
$IV^{\left(d\right)}$	0.9876543210
$C_{0}$	123

**Table 9:** Value ranges of state variables, control parameters, and amounts of perturbation.

State Variables, Control Parameters and Amounts of Perturbation	Value Ranges
${\hat{a}}_{n}^{\left(p\right)}$	[3.6250,3.7500) and [3.8750,4.0)
$\delta_{a}^{\left(p\right)}$	[0,0.1250) and [0.2500,0.3750)
${\hat{a}}_{n}^{\left(d\right)}$	[3.6875,3.7500) and [3.9375,4.0)
$\delta_{a}^{\left(d\right)}$	[0,0.0625) and [0.2500,0.3125)
${\hat{x}}_{n}^{\left(p\right)}$	(0,1)
$\delta_{x}^{\left(p\right)}$	(0,1)
${\hat{x}}_{n}^{\left(d\right)}$	(0,1)
$\delta_{x}^{\left(d\right)}$	(0,1)

**Table 10:** Matrices of bit arrangement.

$Y_{1}^{\left(p\right)}$	[$BIT_{0}$ (1.1) (1.9) (1.2) (1.10) (1.3) (1.11) (1.4) (1.12) (1.5) (1.13) (1.6) (1.14) (1.7) (1.15) (1.8) (1.16) (1.1) (1.9) (1.2) (1.10) (1.3) (1.11) (1.4) (1.12) (1.5) (1.13) (1.6) (1.14) (1.7) (1.15) (1.8) (1.16)]
$iY_{1}^{\left(p\right)}$	[(1.30) (1.18) (1.12) (1.20) (1.16) (1.25) (1.19) (1.13) (1.24) (1.11) (1.26) (1.17) (1.21) (1.24) (1.22) (1.27)]
$Y_{2}^{\left(p\right)}$	[$BIT_{0}$ (1.33) (1.32) (1.31) (1.30) (1.29) (1.28) (1.27) (1.26) (1.25) (1.24) (1.23) (1.22) (1.21) (1.20) (1.19) (1.18) (1.17) (1.16) (1.15) (1.14) (1.13) (1.12) (1.11) (1.10) (1.9) (1.8) (1.7) (1.6) (1.5) (1.4) (1.3) (1.2)]
$Y_{3}^{\left(p\right)}$	[$BIT_{0}$ $BIT_{0}$ $BIT_{0}$ (1.8) $BIT_{0}$ (1.16) (1.7) (1.15) (1.6) (1.14) (1.5) (1.13) (1.4) (1.12) (1.3) (1.11) (1.2) (1.10) (1.1) (1.9) (1.8) (1.16) (1.7) (1.15) (1.6) (1.14) (1.5) (1.13) (1.4) (1.12) (1.3) (1.11) (1.2) (1.10) (1.1) (1.9)]
$Y_{4}^{\left(p\right)}$	[$BIT_{0}$ $BIT_{0}$ $BIT_{0}$ (1.33) $BIT_{0}$ (1.32) (1.31) (1.30) (1.29) (1.28) (1.27) (1.26) (1.25) (1.24) (1.23) (1.22) (1.21) (1.20) (1.19) (1.18) (1.17) (1.16) (1.15) (1.14) (1.13) (1.12) (1.11) (1.10) (1.9) (1.8) (1.7) (1.6) (1.5) (1.4) (1.3) (1.2)]
$Y_{1}^{\left(d\right)}$	[$BIT_{0}$ (1.8) (1.2) (1.5) (1.1) (1.7) (1.2) (1.6) (1.4) (1.5) (1.3) (1.7) (1.8) (1.4) (1.2) (1.7) (1.1) (1.5) (1.3) (1.6) (1.2) (1.4) (1.8) (1.1) (1.3) (1.4) (1.6) (1.5) (1.8) (1.6) (1.1) (1.7) (1.3)]
$iY_{1}^{\left(d\right)}$	[(1.3) (1.8) (1.2) (1.20) (1.16) (1.6) (1.21) (1.30)]
$Y_{2}^{\left(d\right)}$	[$BIT_{0}$ (1.16) (1.11) (1.29) (1.32) (1.18) (1.13) (1.10) (1.7) (1.14) (1.31) (1.4) (1.12) (1.26) (1.5) (1.17) (1.9) (1.22) (1.24) (1.15) (1.21) (1.28) (1.23) (1.6) (1.33) (1.19) (1.8) (1.30) (1.2) (1.3) (1.27) (1.20) (1.25)]
$Y_{3}^{\left(d\right)}$	[$BIT_{0}$ $BIT_{0}$ $BIT_{0}$ (1.8) $BIT_{0}$ $BIT_{0}$ (1.7) (1.6) (1.5) (1.4) (1.3) (1.2) (1.1) (1.1) (1.2) (1.3) (1.4) (1.5) (1.6) (1.7) (1.8) (1.1) (1.2) (1.3) (1.4) (1.5) (1.6) (1.7) (1.8) (1.1) (1.2) (1.3) (1.4) (1.5) (1.6) (1.7) (1.8)]
$Y_{4}^{\left(d\right)}$	[$BIT_{0}$ $BIT_{0}$ $BIT_{0}$ (1.33) $BIT_{0}$ $BIT_{0}$ (1.32) (1.31) (1.30) (1.29) (1.28) (1.27) (1.26) (1.25) (1.24) (1.23) (1.22) (1.21) (1.20) (1.19) (1.18) (1.2) (1.3) (1.4) (1.5) (1.6) (1.7) (1.8) (1.9) (1.10) (1.11) (1.12) (1.13) (1.14) (1.15) (1.16) (1.17)]
$Y_{5}^{\left(d\right)}$	[(1.8) (1.7) (1.6) (1.5) (1.1) (1.2) (1.3) (1.4)]
$iY_{5}^{\left(d\right)}$	[(1.5) (1.6) (1.7) (1.8) (1.4) (1.3) (1.2) (1.1)]

**Table 11:** The number of bits in the secret keys of the permutation and diffusion with perturbation.

The Number of Bits in the Secret Keys				
Scheme		Max. No. of Bits	# of Bits	Sum
**Permutation**	CPP-1	$s_{IV^{\left(p\right)}}$	32	65
		$s_{a^{\left(p\right)}}$	33	
	CPP-2	$s_{IV^{\left(p\right)}}$	32	64
		$s_{a^{\left(p\right)}}$	32	
	CPP-3	$s_{IV^{\left(p\right)}}$	32	64
		$s_{a^{\left(p\right)}}$	32	
**Diffusion**	CD-1	$s_{IV^{\left(d\right)}}$	32	74
		$s_{a^{\left(d\right)}}$	34	
		$s_{C_{0}}$	8	
	CD-2	$s_{IV^{\left(d\right)}}$	32	72
		$s_{a^{\left(d\right)}}$	32	
		$s_{C_{0}}$	8	
	CD-3	$s_{IV^{\left(d\right)}}$	32	72
		$s_{a^{\left(d\right)}}$	32	
		$s_{C_{0}}$	8	

**Table 12:** $\chi^{2}$-test results of original and diffused images.

Perturbation	Round	$\chi^{2}$ Test					
		Lena	Cameraman	House	Peppers	Black	White
Plaintext		30,577.703	161,271.875	299,789.226	36,777.515	16,711,680	16,711,680
On state variable	1	227.977	*313.219*	*316.805*	249.102	*22,864.141*	*27,165.805*
	2	221.000	266.859	*315.852*	251.000	*340.109*	*333.063*
	3	284.180	264.977	273.344	276.367	253.516	259.984
On control parameter	1	284.086	*299.234*	*295.859*	*299.328*	*11,590.945*	*13,372.102*
	2	*299.852*	258.188	286.273	254.219	*308.008*	277.750
	3	**202.352**	241.664	270.242	253.891	238.492	286.703
On both	1	245.086	*402.266*	218.141	220.531	*20,335.578*	*27,947.445*
	2	274.539	278.383	237.500	245.391	*346.742*	*406.969*
	3	249.180	218.383	263.602	231.000	254.031	282.359

**Table 13:** Information entropy of original and diffused images.

Perturbation	Round	IE					
		Lena	Cameraman	House	Peppers	Black	White
Plaintext		7.5691	6.9046	6.4971	7.3785	0	0
On state variable	1	7.9975	7.9966	7.9965	7.9973	*7.7786*	*7.7001*
	2	7.9976	7.9971	7.9965	7.9972	7.9963	7.9963
	3	7.9969	7.9971	7.9970	7.9969	7.9972	7.9971
On control parameter	1	7.9969	7.9967	7.9968	7.9967	*7.8807*	*7.8623*
	2	7.9967	7.9972	7.9969	7.9972	7.9966	7.9969
	3	7.9978	7.9973	7.9970	7.9972	7.9974	7.9968
On both	1	7.9973	7.9956	7.9976	7.9976	*7.8051*	*7.7197*
	2	7.9970	7.9969	7.9974	7.9973	7.9962	7.9955
	3	7.9973	7.9976	7.9971	7.9975	7.9972	7.9969

**Table 14:** Correlation coefficients of permuted, original and diffused Lena image.

CCs of Lena Image					
	Perturbation	Round	Horizontal	Vertical	Diagonal
**Permutation**	On state variable	1	−0.00149	0.00281	0.00459
		2	0.00636	−0.00316	0.00186
		3	0.00104	0.00567	−0.00178
	On control parameter	1	0.00404	0.00186	−0.00447
		2	−0.00317	0.00474	0.00226
		3	0.00432	−0.00125	−0.00538
	On both	1	−0.00177	0.00019	0.00383
		2	−0.00158	−0.00042	0.00238
		3	0.00050	−0.00266	0.00627
Plaintext			0.93998	0.96934	0.91793
**Diffusion**	On state variable	1	0.00400	−0.00131	−0.00288
		2	−0.00260	*0.01085*	0.00013
		3	0.00598	0.00835	−0.00248
	On control parameter	1	0.00102	−0.00715	−0.00139
		2	0.00121	0.00446	0.00829
		3	0.00150	−0.00272	−0.00231
	On both	1	0.00034	0.00272	0.00070
		2	−0.00829	0.00105	−0.00458
		3	0.00211	−0.00063	−0.00040

**Table 15:** Correlation coefficients of permuted, original and diffused Cameraman image.

CCs of Cameraman Image					
	Perturbation	Round	Horizontal	Vertical	Diagonal
**Permutation**	On state variable	1	−0.00264	0.00256	−0.00015
		2	0.00304	−0.00099	−0.00230
		3	0.00295	−0.00813	−0.00334
	On control parameter	1	−0.00007	−0.00572	0.00292
		2	−0.00148	0.00059	−0.00221
		3	−0.00002	−0.00730	−0.00038
	On both	1	0.00016	0.00699	−0.00164
		2	0.00109	−0.00307	−0.00197
		3	0.00142	0.00110	−0.00177
Plaintext			0.91957	0.95494	0.89619
**Diffusion**	On state variable	1	−0.00179	0.00109	0.00259
		2	−0.00550	0.00317	−0.00412
		3	−0.00091	−0.00021	−0.00421
	On control parameter	1	0.00127	−0.00033	0.00351
		2	−0.00086	−0.00360	0.00576
		3	0.00621	−0.00220	0.00038
	On both	1	−0.00147	−0.00302	−0.00257
		2	0.00396	−0.00156	0.00449
		3	−0.00305	0.00035	−0.00381

**Table 16:** Correlation coefficients of permuted, original and diffused House image.

CCs of House Image					
	Perturbation	Round	Horizontal	Vertical	Diagonal
**Permutation**	On state variable	1	−0.00188	−0.00469	0.00974
		2	−0.00069	0.00607	−0.00130
		3	−0.00218	−0.00551	−0.00226
	On control parameter	1	0.00321	0.00240	−0.00824
		2	0.00351	0.00244	−0.00432
		3	−0.00174	−0.00843	−0.00204
	On both	1	−0.00305	0.00655	−0.00033
		2	−0.00494	0.00361	0.00058
		3	−0.00776	−0.00395	−0.00247
Plaintext			0.97807	0.96528	0.94835
**Diffusion**	On state variable	1	−0.00276	−0.00311	0.00080
		2	−0.00514	−0.00252	−0.00372
		3	0.00223	0.00283	0.00318
	On control parameter	1	−0.00158	0.00838	−0.00022
		2	0.00250	0.00110	−0.00296
		3	−0.00127	−0.00378	−0.00435
	On both	1	0.00623	0.00086	0.00006
		2	−0.00305	0.00285	0.00833
		3	0.00254	0.00117	0.00283

**Table 17:** Correlation coefficients of permuted, original and diffused Peppers image.

CCs of Peppers Image					
	Perturbation	Round	Horizontal	Vertical	Diagonal
**Permutation**	On state variable	1	0.00126	0.00560	−0.00196
		2	−0.00378	0.00150	0.00998
		3	0.00630	0.00290	−0.00124
	On control parameter	1	−0.00130	0.00101	−0.00116
		2	−0.00210	0.00167	−0.00204
		3	0.00610	0.00559	−0.00486
	On both	1	−0.00391	−0.00237	0.00564
		2	0.00463	0.00445	0.00077
		3	−0.00186	0.00124	−0.00264
Plaintext			0.94777	0.94819	0.90359
**Diffusion**	On state variable	1	0.00312	0.00428	0.00276
		2	−0.00706	−0.00263	−0.00587
		3	*0.01129*	0.00016	0.00548
	On control parameter	1	−0.00205	0.00658	0.00358
		2	−0.00166	0.00271	0.00156
		3	−0.00023	−0.00465	−0.00167
	On both	1	0.00629	0.00720	−0.00560
		2	−0.00117	0.00391	0.00134
		3	0.00265	−0.00378	0.00388

**Table 18:** Correlation coefficients of diffused Black image.

CCs of Black Image					
	Perturbation	Round	Horizontal	Vertical	Diagonal
	Plaintext		NaN	NaN	NaN
**Diffusion**	On state variable	1	*−0.01423*	*−0.01140*	0.00404
		2	0.00063	0.00347	−0.00619
		3	−0.00670	0.00135	−0.00505
	On control parameter	1	0.00736	0.00133	−0.00894
		2	0.00318	−0.00488	−0.00340
		3	−0.00170	0.00001	0.00363
	On both	1	−0.00087	0.00442	*0.01184*
		2	−0.00169	−0.00663	0.00323
		3	0.00367	−0.00358	−0.00115

**Table 19:** Correlation coefficients of diffused White image.

CCs of White Image					
	Perturbation	Round	Horizontal	Vertical	Diagonal
	Plaintext		NaN	NaN	NaN
**Diffusion**	On state variable	1	0.00650	0.07976	0.00418
		2	−0.00068	0.00049	−0.00469
		3	−0.00545	−0.00087	0.00173
	On control parameter	1	*0.02599*	*0.01076*	−0.00367
		2	0.00802	0.00179	−0.00064
		3	−0.00398	0.00487	0.00032
	On both	1	*0.01336*	−0.04708	0.00202
		2	0.00213	*−0.01066*	0.00210
		3	−0.00394	0.00377	−0.00268

**Table 20:** The values of $\Delta_{K}$ for Cdr.

	ΔK	Amount in Binary	Value of Tolerance
**Permutation**	$\Delta K_{IV}^{\left(CPP-1\right)}$	0.00000000000000000000000000000001	$2^{-32}$
	$\Delta K_{a}^{\left(CPP-2\right)}$	0.0000000000000000000000000000000001	$2^{-34}$
	$\Delta K_{IV}^{\left(CPP-3\right)}$	0.00000000000000000000000000000001	$2^{-32}$
	$\Delta K_{a}^{\left(CPP-3\right)}$	0.0000000000000000000000000000000001	$2^{-34}$
**Diffusion**	$\Delta K_{IV}^{\left(CD-1\right)}$	0.00000000000000000000000000000001	$2^{-32}$
	$\Delta K_{a}^{\left(CD-2\right)}$	0.00000000000000000000000000000000001	$2^{-35}$
	$\Delta K_{IV}^{\left(CD-3\right)}$	0.00000000000000000000000000000001	$2^{-32}$
	$\Delta K_{a}^{\left(CD-3\right)}$	0.00000000000000000000000000000000001	$2^{-35}$
	$\Delta K_{C_{0}}$	00000001	1

**Table 21:** Ciphertext difference rates of permuted and diffused Lena image.

CDRs of Lena Image					
	Perturbation	Round	Cdr_IV	Cdr_a	$\mathrm{Cdr}\_C_{0}$
**Permutation**	On state variable	1	*81.876*	*63.679*	-
		2	92.301	*84.467*	-
		3	96.131	92.413	-
	On control parameter	1	99.384	*80.531*	-
		2	99.424	91.125	-
		3	99.439	95.380	-
	On both	1	99.177	*81.901*	-
		2	99.414	92.160	-
		3	99.427	96.044	-
**Diffusion**	On state variable	1	99.485	99.516	99.528
		2	99.591	99.583	99.607
		3	99.607	99.599	99.603
	On control parameter	1	99.530	99.546	99.509
		2	99.628	99.643	99.638
		3	99.617	99.609	99.622
	On both	1	99.496	99.459	99.441
		2	99.602	99.591	99.616
		3	99.640	99.615	99.605

**Table 22:** Ciphertext difference rates of permuted and diffused Cameraman image.

CDRs of Cameraman Image					
	Perturbation	Round	Cdr_IV	Cdr_a	$\mathrm{Cdr}\_C_{0}$
**Permutation**	On state variable	1	*81.145*	*63.070*	-
		2	91.579	*83.722*	-
		3	95.350	91.621	-
	On control parameter	1	98.576	*79.908*	-
		2	98.669	90.435	-
		3	98.658	94.621	-
	On both	1	98.399	*81.179*	-
		2	98.618	91.403	-
		3	98.583	95.308	-
**Diffusion**	On state variable	1	99.506	99.501	99.485
		2	99.598	99.541	99.601
		3	99.622	99.590	99.608
	On control parameter	1	99.550	99.565	99.550
		2	99.566	99.567	99.608
		3	99.610	99.593	99.622
	On both	1	99.471	99.494	99.489
		2	99.609	99.610	99.635
		3	99.601	99.624	99.559

**Table 23:** Ciphertext difference rates of permuted and diffused House image.

CDRs of House Image					
	Perturbation	Round	Cdr_IV	Cdr_a	$\mathrm{Cdr}\_C_{0}$
**Permutation**	On state variable	1	*80.491*	*62.514*	-
		2	90.806	*83.016*	-
		3	94.501	90.883	-
	On control parameter	1	97.781	*79.177*	-
		2	97.842	*89.648*	-
		3	97.790	93.845	-
	On both	1	97.582	*80.533*	-
		2	97.720	90.611	-
		3	97.836	94.540	-
**Diffusion**	On state variable	1	99.505	99.526	99.534
		2	99.593	99.598	99.626
		3	99.611	99.619	99.609
	On control parameter	1	99.539	99.547	99.546
		2	99.598	99.609	99.621
		3	99.615	99.603	99.630
	On both	1	99.446	99.517	99.483
		2	99.602	99.649	99.628
		3	99.609	99.601	99.616

**Table 24:** Ciphertext difference rates of permuted and diffused Peppers image.

CDRs of Peppers Image					
	Perturbation	Round	Cdr_IV	Cdr_a	$\mathrm{Cdr}\_C_{0}$
**Permutation**	On state variable	1	*81844*	*63.633*	-
		2	92.255	*84.453*	-
		3	96.104	92.378	-
	On control parameter	1	99.412	*80.538*	-
		2	99.395	91.091	-
		3	99.376	95.348	-
	On both	1	99.132	*81.837*	-
		2	99.332	92.104	-
		3	99.342	95.979	-
**Diffusion**	On state variable	1	99.519	99.548	99.506
		2	99.621	99.611	99.635
		3	99.590	99.593	99.596
	On control parameter	1	99.539	99.532	99.550
		2	99.612	99.570	99.601
		3	99.628	99.609	99.646
	On both	1	99.525	99.506	99.492
		2	99.614	99.593	99.605
		3	99.607	99.581	99.622

**Table 25:** Ciphertext difference rates of diffused Black image.

CDRs of Black Image					
	Diffusion	Round	Cdr_IV	Cdr_a	$\mathrm{Cdr}\_C_{0}$
**Permutation**	On state variable	1	99.464	99.535	99.536
		2	99.636	99.631	99.602
		3	99.636	99.612	99.629
	On control parameter	1	99.596	99.561	99.545
		2	99.629	99.605	99.577
		3	99.610	99.612	99.608
	On both	1	99.505	99.490	99.496
		2	99.621	99.611	99.612
		3	99.612	99.596	99.619

**Table 26:** Ciphertext difference rates of diffused White image.

CDRs of White Image					
	Diffusion	Round	Cdr_IV	Cdr_a	$\mathrm{Cdr}\_C_{0}$
**Permutation**	On state variable	1	99.526	99.307	99.551
		2	99.584	99.601	99.602
		3	99.635	99.597	99.608
	On control parameter	1	99.601	99.532	99.548
		2	99.608	99.596	99.609
		3	99.605	99.610	99.634
	On both	1	99.652	99.487	99.495
		2	99.587	99.596	99.625
		3	99.583	99.600	99.616

**Table 27:** Sensitivity to secret keys: Lena image.

Sensitivity to Secret Keys: Lena Image								
	Permutation on	Round	$\Delta K_{\mathrm{IV}}$		$\Delta K_{a}$		$\Delta K_{C_{0}}$	
			NPCR (%)	UACI (%)	NPCR (%)	UACI (%)	NPCR (%)	UACI (%)
**Permutation**	On state variable	1	*98.920*	*23.359*	*62.524*	*14.660*	-	-
		2	99.370	*23.433*	*83.701*	*19.769*	-	-
		3	99.393	*23.444*	*91.956*	*21.718*	-	-
	On control parameter	1	99.361	*23.383*	*61.656*	*14.564*	-	-
		2	99.437	*23.483*	*82.838*	*19.531*	-	-
		3	99.448	*23.497*	*91.330*	*21.544*	-	-
	On both	1	*98.921*	*23.393*	*64.369*	*15.141*	-	-
		2	99.406	*23.446*	*84.898*	*19.911*	-	-
		3	99.414	*23.416*	*92.648*	*21.802*	-	-
**Diffusion**	On state variable	1	99.474	*32.444*	99.506	*32.512*	99.532	*32.560*
		2	99.548	33.392	99.577	33.485	99.582	33.557
		3	99.585	33.326	99.622	33.474	99.606	33.394
	On control parameter	1	99.513	*32.449*	99.550	*32.376*	99.529	*32.437*
		2	99.641	33.510	99.629	33.430	99.683	33.407
		3	99.614	33.447	99.593	33.573	99.606	33.468
	On both	1	99.468	*32.089*	99.446	*31.995*	99.426	*31.887*
		2	99.609	33.470	99.579	33.375	99.612	33.586
		3	99.638	33.480	99.623	33.533	99.620	33.416

**Table 28:** Sensitivity to secret keys: Cameraman image.

Sensitivity to Secret Keys: Cameraman Image								
	Permutation on	Round	$\Delta K_{\mathrm{IV}}$		$\Delta K_{a}$		$\Delta K_{C_{0}}$	
			NPCR (%)	UACI (%)	NPCR (%)	UACI (%)	NPCR (%)	UACI (%)
**Permutation**	On state variable	1	*98.071*	*27.877*	*61.920*	*17.583*	-	-
		2	*98.602*	*27.971*	*82.889*	*23.405*	-	-
		3	*98.647*	*28.020*	*91.190*	*25.839*	-	-
	On control parameter	1	*98.555*	*27.962*	*61.220*	*17.229*	-	-
		2	*98.674*	*28.049*	*82.205*	*23.121*	-	-
		3	*98.659*	*28.053*	*90.585*	*25.709*	-	-
	On both	1	*98.160*	*27.717*	*63.721*	*18.064*	-	-
		2	*98.653*	*27.902*	*84.224*	*23.973*	-	-
		3	*98.496*	*28.072*	*91.945*	*26.125*	-	-
**Diffusion**	On state variable	1	99.461	*32.415*	99.487	*32.640*	99.500	*32.382*
		2	99.600	33.496	99.567	33.348	99.609	33.454
		3	99.652	33.332	99.628	33.155	99.565	33.427
	On control parameter	1	99.542	*32.622*	99.539	*32.430*	99.516	*32.513*
		2	99.554	33.576	99.545	33.477	99.628	33.544
		3	99.608	33.472	99.616	33.351	99.617	33.427
	On both	1	99.435	*31.799*	99.490	*32.057*	99.442	*32.075*
		2	99.617	33.435	99.614	33.663	99.605	33.385
		3	99.587	33.604	99.625	33.548	99.548	33.514

**Table 29:** Sensitivity to secret keys: House image.

Sensitivity to Secret Keys: House Image								
	Permutation on	Round	$\Delta K_{\mathrm{IV}}$		$\Delta K_{a}$		$\Delta K_{C_{0}}$	
			NPCR (%)	UACI (%)	NPCR (%)	UACI (%)	NPCR (%)	UACI (%)
**Permutation**	On state variable	1	*97.379*	*20.122*	*61.424*	*12.763*	-	-
		2	*97.783*	*20.326*	*82.204*	*17.091*	-	-
		3	*97.697*	*20.252*	*90.462*	*18.823*	-	-
	On control parameter	1	*97.836*	*20.289*	*60.628*	*12.628*	-	-
		2	*97.862*	*20.341*	*81.473*	*16.876*	-	-
		3	*97.772*	*20.339*	*89.882*	*18.700*	-	-
	On both	1	*97.325*	*20.172*	*63.226*	*13.152*	-	-
		2	*97.707*	*20.219*	*83.489*	*17.351*	-	-
		3	*97.765*	*20.353*	*91.173*	*18.894*	-	-
**Diffusion**	On state variable	1	99.516	*32.538*	99.526	*32.392*	99.535	*32.441*
		2	99.553	33.448	99.585	33.333	99.619	33.575
		3	99.614	33.525	99.640	33.423	99.619	33.477
	On control parameter	1	99.498	*32.336*	99.559	*32.508*	99.559	*32.399*
		2	99.574	33.368	99.631	33.329	99.619	33.619
		3	99.614	33.439	99.605	33.383	99.623	33.398
	On both	1	99.455	*32.238*	99.507	*31.965*	99.468	*32.009*
		2	99.591	33.318	99.634	33.481	99.629	33.376
		3	99.609	33.507	99.600	33.478	99.631	33.529

**Table 30:** Sensitivity to secret keys: Peppers image.

Sensitivity to Secret Keys: House Image								
	Permutation on	Round	$\Delta K_{\mathrm{IV}}$		$\Delta K_{a}$		$\Delta K_{C_{0}}$	
			NPCR (%)	UACI (%)	NPCR (%)	UACI (%)	NPCR (%)	UACI (%)
**Permutation**	On state variable	1	*98.917*	*23.692*	*62.494*	*14.903*	-	-
		2	99.292	*23.818*	*83.687*	*20.009*	-	-
		3	99.377	*23.869*	*91.925*	*21.990*	-	-
	On control parameter	1	99.405	*23.712*	*61.658*	*14.658*	-	-
		2	99.446	*23.858*	*82.838*	*19.715*	-	-
		3	99.379	*23.881*	*91.324*	*21.848*	-	-
	On both	1	*98.897*	*23.673*	*64.308*	*15.397*	-	-
		2	99.313	*23.774*	*84.856*	*20.233*	-	-
		3	99.353	*23.813*	*92.627*	*22.141*	-	-
**Diffusion**	On state variable	1	99.539	*32.535*	99.524	*32.530*	99.489	*32.251*
		2	99.599	33.483	99.591	33.446	99.655	33.359
		3	99.617	33.597	99.629	33.506	99.564	33.451
	On control parameter	1	99.536	*32.516*	99.529	*32.735*	99.541	*32.409*
		2	99.602	33.342	99.593	33.397	99.583	33.332
		3	99.651	33.562	99.616	33.392	99.641	33.527
	On both	1	99.510	*32.037*	99.493	*31.951*	99.469	*32.133*
		2	99.597	33.601	99.594	33.534	99.625	33.599
		3	99.608	33.468	99.571	33.328	99.612	33.412

**Table 31:** Sensitivity to secret keys: Black image.

Sensitivity to Secret Keys: House Image								
	Permutation on	Round	$\Delta K_{\mathrm{IV}}$		$\Delta K_{a}$		$\Delta K_{C_{0}}$	
			NPCR (%)	UACI (%)	NPCR (%)	UACI (%)	NPCR (%)	UACI (%)
**Permutation**	On state variable	1	99.458	33.840	99.475	34.093	99.655	34.720
		2	99.631	33.481	99.634	33.311	99.588	33.423
		3	99.658	33.527	99.628	33.464	99.635	33.485
	On control parameter	1	99.574	*32.691*	99.550	*32.792*	99.536	*32.796*
		2	99.643	33.532	99.612	33.557	99.590	33.438
		3	99.593	33.447	99.626	33.466	99.611	33.541
	On both	1	99.498	33.002	99.512	33.075	99.501	*32.825*
		2	99.603	33.436	99.609	33.444	99.608	33.431
		3	99.612	33.476	99.609	33.337	99.614	33.361

**Table 32:** Sensitivity to secret keys: White image.

Sensitivity to Secret Keys: House Image								
	Permutation on	Round	$\Delta K_{\mathrm{IV}}$		$\Delta K_{a}$		$\Delta K_{C_{0}}$	
			NPCR (%)	UACI (%)	NPCR (%)	UACI (%)	NPCR (%)	UACI (%)
**Permutation**	On state variable	1	99.490	33.649	99.503	32.983	99.526	33.594
		2	99.580	33.268	99.588	33.375	99.605	33.362
		3	99.619	33.449	99.614	33.587	99.611	33.418
	On control parameter	1	99.579	*32.484*	99.539	*32.620*	99.574	*32.542*
		2	99.553	33.582	99.564	33.500	99.622	33.480
		3	99.606	33.412	99.620	33.577	99.620	33.608
	On both	1	99.715	33.137	99.490	*32.962*	99.486	*32.627*
		2	99.591	33.239	99.579	33.613	99.619	33.342
		3	99.580	33.431	99.596	33.449	99.602	33.627

**Table 33:** Quality of permutation based on MSE and PSNR.

Perturbation	Round	Lena		Cameraman		House		Peppers	
		MSE	PSNR	MSE	PSNR	MSE	PSNR	MSE	PSNR
On state variable	1	$5.498\times 10^{3}$	10.729	9.292 $\times 10^{3}$	8.450	4.180 $\times 10^{3}$	11.919	5.658 $\times 10^{3}$	10.604
	2	5.448 $\times 10^{3}$	10.769	9.420 $\times 10^{3}$	8.390	4.208 $\times 10^{3}$	11.890	5.634 $\times 10^{3}$	10.622
	3	5.475 $\times 10^{3}$	10.747	9.359 $\times 10^{3}$	8.419	4.200 $\times 10^{3}$	11.898	5.636 $\times 10^{3}$	10.621
On control parameter	1	5.471 $\times 10^{3}$	10.750	9.422 $\times 10^{3}$	8.389	4.184 $\times 10^{3}$	11.915	5.679 $\times 10^{3}$	10.588
	2	5.461 $\times 10^{3}$	10.758	9.355 $\times 10^{3}$	8.421	4.220 $\times 10^{3}$	11.877	5.642 $\times 10^{3}$	10.617
	3	5.481 $\times 10^{3}$	10.742	9.446 $\times 10^{3}$	8.378	4.235 $\times 10^{3}$	11.862	5.666 $\times 10^{3}$	10.598
On both	1	5.457 $\times 10^{3}$	10.762	9.361 $\times 10^{3}$	8.418	4.164 $\times 10^{3}$	11.935	5.648 $\times 10^{3}$	10.611
	2	5.439 $\times 10^{3}$	10.776	9.398 $\times 10^{3}$	8.401	4.181 $\times 10^{3}$	11.918	5.641 $\times 10^{3}$	10.617
	3	5.456 $\times 10^{3}$	10.762	9.409 $\times 10^{3}$	8.395	4.243 $\times 10^{3}$	11.854	5.642 $\times 10^{3}$	10.616

**Table 34:** Quality of diffusion based on MSE, PSNR, NPCR and UACI.

Perturbation	Round	Lena				Cameraman				House			
		MSE	PSNR	NPCR	UACI	MSE	PSNR	NPCR	UACI	MSE	PSNR	NPCR	UACI
On state variable	1	9.053 $\times 10^{3}$	8.563	99.669	33.507	1.171 $\times 10^{4}$	7.447	99.530	32.436	7.673 $\times 10^{3}$	9.281	99.519	32.343
	2	8.950 $\times 10^{3}$	8.613	99.582	33.446	1.174 $\times 10^{4}$	7.435	99.640	33.405	7.720 $\times 10^{3}$	9.255	99.611	33.529
	3	9.008 $\times 10^{3}$	8.585	99.596	33.343	1.174 $\times 10^{4}$	7.436	99.612	33.423	7.734 $\times 10^{3}$	9.247	99.634	33.464
On control parameter	1	9.017 $\times 10^{3}$	8.580	99.626	33.515	1.181 $\times 10^{4}$	7.408	99.538	32.625	7.617 $\times 10^{3}$	9.313	99.599	32.417
	2	9.028 $\times 10^{3}$	8.575	99.603	33.475	1.180 $\times 10^{4}$	7.411	99.651	33.663	7.716 $\times 10^{3}$	9.257	99.544	33.480
	3	9.027 $\times 10^{3}$	8.575	99.609	33.412	1.178 $\times 10^{4}$	7.420	99.594	33.377	7.641 $\times 10^{3}$	9.299	99.596	33.475
On both	1	9.036 $\times 10^{3}$	8.571	99.602	33.471	1.172 $\times 10^{4}$	7.443	99.458	32.003	7.635 $\times 10^{3}$	9.303	99.486	32.144
	2	9.049 $\times 10^{3}$	8.565	99.577	33.485	1.159 $\times 10^{4}$	7.490	99.597	33.603	7.750 $\times 10^{3}$	9.238	99.617	33.537
	3	9.078 $\times 10^{3}$	8.551	99.596	33.557	1.173 $\times 10^{4}$	7.438	99.637	33.425	7.706 $\times 10^{3}$	9.262	99.658	33.567
**Perturbation**	**Round**	**Peppers**				**Black**				**White**			
		**MSE**	**PSNR**	**NPCR**	**UACI**	**MSE**	**PSNR**	**NPCR**	**UACI**	**MSE**	**PSNR**	**NPCR**	**UACI**
On state variable	1	8.388 $\times 10^{3}$	8.894	99.539	32.344	2.842 $\times 10^{4}$	3.595	99.638	34.519	2.783 $\times 10^{4}$	3.685	99.550	33.651
	2	8.363 $\times 10^{3}$	8.907	99.608	33.370	2.160 $\times 10^{4}$	4.785	99.620	33.408	2.158 $\times 10^{4}$	4.790	99.608	33.395
	3	8.304 $\times 10^{3}$	8.938	99.614	33.498	2.174 $\times 10^{4}$	4.758	99.616	33.457	2.177 $\times 10^{4}$	4.753	99.616	33.535
On control parameter	1	8.331 $\times 10^{3}$	8.924	99.545	32.445	2.690 $\times 10^{4}$	3.834	99.556	32.726	2.692 $\times 10^{4}$	3.829	99.571	32.695
	2	8.307 $\times 10^{3}$	8.936	99.605	33.588	2.157 $\times 10^{4}$	4.791	99.625	33.424	2.175 $\times 10^{4}$	4.756	99.608	33.538
	3	8.292 $\times 10^{3}$	8.944	99.585	33.350	2.172 $\times 10^{4}$	4.762	99.622	33.489	2.173 $\times 10^{4}$	4.761	99.631	33.366
On both	1	8.400 $\times 10^{3}$	8.888	99.492	32.118	2.797 $\times 10^{4}$	3.663	99.464	32.788	2.844 $\times 10^{4}$	3.592	99.567	32.127
	2	8.317 $\times 10^{3}$	8.931	99.645	33.318	2.162 $\times 10^{4}$	4.781	99.585	33.380	2.188 $\times 10^{4}$	4.730	99.634	33.577
	3	8.337 $\times 10^{3}$	8.921	99.605	33.522	2.178 $\times 10^{4}$	4.751	99.619	33.314	2.168 $\times 10^{4}$	4.771	99.583	33.539

**Table 35:** Quality of inverse diffusion based on MSE, PSNR, NPCR and UACI.

Perturbation	Round	Lena				Cameraman				House			
		MSE	PSNR	NPCR	UACI	MSE	PSNR	NPCR	UACI	MSE	PSNR	NPCR	UACI
On state variable	1	9.006 $\times 10^{3}$	8.586	99.640	30.508	1.174 $\times 10^{4}$	7.436	99.526	34.751	7.766 $\times 10^{3}$	9.229	99.507	28.680
	2	9.076 $\times 10^{3}$	8.552	99.616	30.638	1.174 $\times 10^{4}$	7.433	99.606	34.740	7.661 $\times 10^{3}$	9.288	99.588	28.407
	3	9.033 $\times 10^{3}$	8.573	99.612	30.610	1.168 $\times 10^{4}$	7.457	99.600	34.599	7.652 $\times 10^{3}$	9.293	99.567	28.457
On control parameter	1	9.064 $\times 10^{3}$	8.558	99.602	30.650	1.172 $\times 10^{4}$	7.440	99.527	34.648	7.758 $\times 10^{3}$	9.233	99.542	28.635
	2	8.993 $\times 10^{3}$	8.592	99.617	30.439	1.166 $\times 10^{4}$	7.465	99.628	34.646	7.738 $\times 10^{3}$	9.244	99.660	28.643
	3	8.991 $\times 10^{3}$	8.593	99.611	30.463	1.168 $\times 10^{4}$	7.457	99.619	34.590	7.685 $\times 10^{3}$	9.275	99.643	28.482
On both	1	9.038 $\times 10^{3}$	8.570	99.593	30.608	1.168 $\times 10^{4}$	7.456	99.484	34.607	7.680 $\times 10^{3}$	9.277	99.497	28.482
	2	9.027 $\times 10^{3}$	8.575	99.614	30.560	1.164 $\times 10^{4}$	7.473	99.602	34.547	7.683 $\times 10^{3}$	9.275	99.641	28.476
	3	9.112 $\times 10^{3}$	8.535	99.611	30.758	1.175 $\times 10^{4}$	7.431	99.614	34.712	7.687 $\times 10^{3}$	9.273	99.596	28.486
**Perturbation**	**Round**	**Peppers**				**Black**				**White**			
		**MSE**	**PSNR**	**NPCR**	**UACI**	**MSE**	**PSNR**	**NPCR**	**UACI**	**MSE**	**PSNR**	**NPCR**	**UACI**
On state variable	1	8.267 $\times 10^{3}$	8.958	99.599	29.396	2.167 $\times 10^{4}$	4.772	99.515	49.902	2.164 $\times 10^{4}$	4.778	99.469	49.836
	2	8.338 $\times 10^{3}$	8.920	99.521	29.474	2.155 $\times 10^{4}$	4.796	99.583	49.718	2.179 $\times 10^{4}$	4.748	99.634	50.126
	3	8.304 $\times 10^{3}$	8.938	99.583	29.420	2.170 $\times 10^{4}$	4.767	99.593	49.954	2.174 $\times 10^{4}$	4.758	99.609	49.990
On control parameter	1	8.321 $\times 10^{3}$	8.929	99.583	29.458	2.159 $\times 10^{4}$	4.788	99.593	49.761	2.159 $\times 10^{4}$	4.788	99.545	49.853
	2	8.305 $\times 10^{3}$	8.937	99.600	29.470	2.170 $\times 10^{4}$	4.767	99.603	49.964	2.163 $\times 10^{4}$	4.779	99.643	49.900
	3	8.302 $\times 10^{3}$	8.939	99.622	29.465	2.173 $\times 10^{4}$	4.761	99.640	50.021	2.178 $\times 10^{4}$	4.750	99.619	50.060
On both	1	8.304 $\times 10^{3}$	8.938	99.498	29.425	2.172 $\times 10^{4}$	4.762	99.492	50.025	2.148 $\times 10^{4}$	4.810	99.469	49.646
	2	8.298 $\times 10^{3}$	8.941	99.599	29.396	2.159 $\times 10^{4}$	4.789	99.626	49.821	2.173 $\times 10^{4}$	4.760	99.631	50.057
	3	8.322 $\times 10^{3}$	8.928	99.643	29.464	2.170 $\times 10^{4}$	4.766	99.611	49.968	2.175 $\times 10^{4}$	4.756	99.652	50.046
